# *Coicis Semen* for the treatment of malignant tumors of the female reproductive system: A review of traditional Chinese medicinal uses, phytochemistry, pharmacokinetics, and pharmacodynamics

**DOI:** 10.3389/fphar.2023.1129874

**Published:** 2023-02-23

**Authors:** Xue Pan, Qian Shen, Chuanlong Zhang, Xiyuan Zhang, Yi Li, Zhuo Chang, Bo Pang

**Affiliations:** ^1^ Post-doctoral Mobile Station, Guang’anmen Hospital, China Academy of Chinese Medical Sciences, Beijing, China; ^2^ International Medical Department, Guang’anmen Hospital, China Academy of Chinese Medical Sciences, Beijing, China; ^3^ Graduate School of Beijing University of Chinese Medicine, Beijing, China; ^4^ Heilongjiang University of Chinese Medicine, Harbin, Heilongjiang, China

**Keywords:** clinical trial review, coicis semen, female reproductive system malignancy, pharmacodynamics, pharmacokinetics, phytochemistry

## Abstract

*Coicis* *Semen* is an important food product and traditional Chinese medicine (TCM) derived from the dried and mature seeds of *Coix lacryma-jobi L.*var.*ma-yuen* (Roman.) Stapf. An increasing number of studies have investigated its use, either alone or in combination with other botanical drugs, to treat female reproductive system malignancies, and its pharmacological effects have been confirmed clinically. This review aims to provide an overview of *Coicis Semen*’s historical role in treating female reproductive system malignancies based on TCM theory, to summarize clinical trials results, and to analyze information pertaining to the main phytochemical components, pharmacokinetics, related anti-cancer pharmacological effects, and toxicology of *Coicis Semen*. Information on *Coicis Semen* was collected from internationally accepted scientific databases. Seventy-four clinical trials were identified that used *Coicis Semen* in combination with other Chinese medicine to treat female reproductive system malignancies, most of which demonstrated good anti-tumor efficacy and few adverse reactions. To date, more than 80 individual compounds have been isolated from this botanical drug. In terms of anti-tumor effects, Coix seed oil has been studied the most. Pharmacokinetic data suggest that the active ingredients in *Coicis Semen* are widely distributed after administration, and *Coicis Semen* and its active compounds play a beneficial role in treating female reproductive system malignancies. Mechanistically, the anti-cancer effects may be related to inhibition of tumor cell proliferation and promotion of apoptosis, inhibition of tumor angiogenesis, suppression of the chronic inflammatory microenvironment of tumors, modulation of immune function, and regulation of the female reproductive system. Most acute toxicity and genotoxicity studies have shown that *Coicis Semen* is non-toxic. However, the existing studies have many limitations, and the future research direction should emphasize 1) the relationship between drug concentration and pharmacological action as well as toxicity; 2) the structural modification or the synthesis of analogues led by the active ingredients of *Coicis Semen* to enhance pharmacological activities and bioavailability; 3) accurately revealing the anti-cancer pharmacological effects of *Coicis Semen* and its compounds through multi-omics technology. We hope that this review can determine future directions and inform novel drug development for treating female reproductive malignancies.

## 1 Introduction

In recent years, changes in human reproductive behavior, lifestyles, nutritional conditions, and the surrounding environment have gradually increased the incidence of and mortality from tumors affecting the female reproductive system. The latest epidemiological data have shown that cervical cancer, cancer of the uterine corpus (which predominantly includes adenocarcinomas originating in the endometrium and some other rarer cancers, such as sarcomas) and ovarian cancer, are three of the most common malignant tumors of the female reproductive system and are among the top 10 such tumors worldwide in terms of incidence. The incidence rate ratio of these three cancers was shown to be 28.6%, and they were responsible for 646,000 deaths in 2020, with an increasing trend observed year-by-year (Weiderpass and Labrèche, 2012; Sung et al., 2021). Malignant tumors of the female reproductive system have become an important public health problem; thus, it is becoming increasingly important to develop effective prevention and treatment strategies. Although some progress has been made, conventional treatments often cannot effectively relieve the clinical symptoms and signs of malignant tumors, and the clinical efficacy of such therapies is greatly affected by the need to minimize associated side effects (Small et al., 2017; Stewart et al., 2019; Makker et al., 2021). Traditional Chinese medicine (TCM) has certain advantages over conventional Western treatments, as it represents a more holistic multi-target option that is cheap and safe. In recent years, these natural products have gradually attracted the attention of researchers.

**FIGURE 1:**
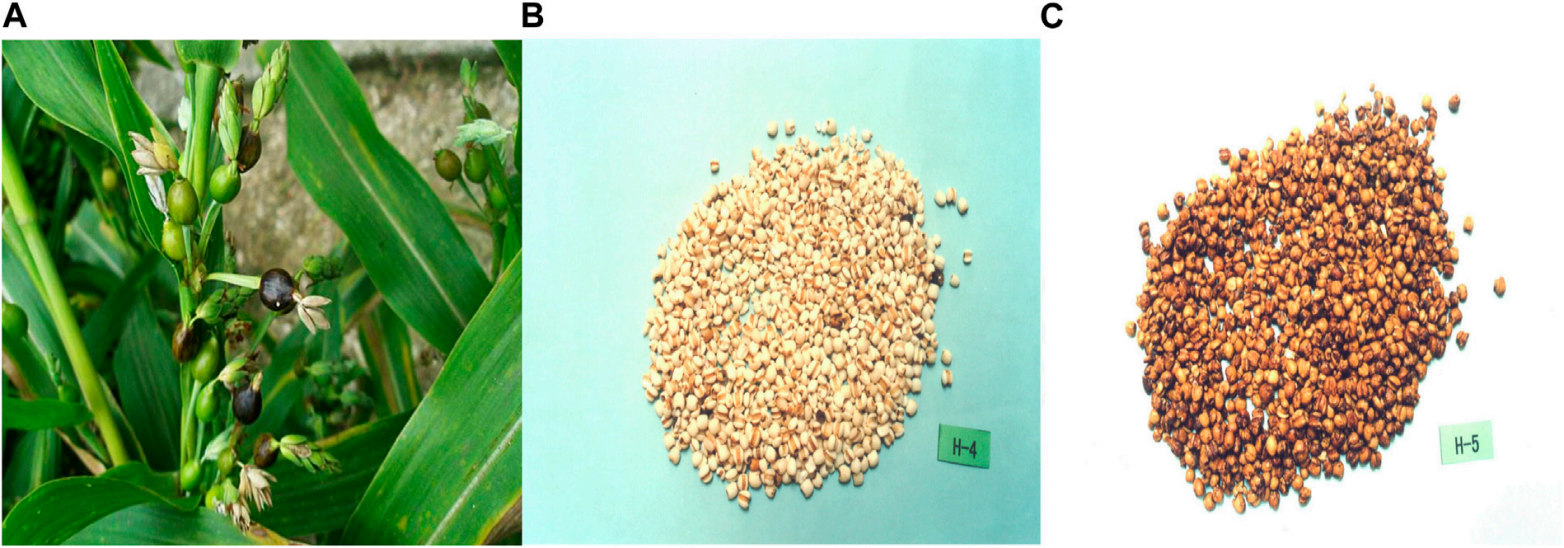
Representative images of *Coicis Semen.* **(A)**, Raw *Coicis Semen.* **(B)**, Fried *Coicis Semen.* **(C)**, Seed kernels of *Coicis Semen,* which are broadly ovate or oblong in shape, 4–8 mm in length, and 3–6 mm in width. Pictures are from the Shanghai Institute of Organic Chemistry of the Chemical Abstracts Service Online Database [CAS(DB/OL)], accessible at: http://www.organchem.csdb.cn.[1978-2021].

*Coicis Semen* (Figure 1), a food product that is used in TCM, is derived from the dried and mature seeds of *Coix lacryma-jobi L.*var.*ma-yuen* (Roman.) Stapf. The use of Chinese *Coicis Semen* can be traced back to the Neolithic Age, and it is distributed in many regions, mainly in Fujian, Guizhou, and Hunan. Some properties of *Coicis Semen* were first recorded in Shennong’s Classic Materia Medica (Shennong Bencao Jing, Han Dynasty, 282 CE), which described its taste, efficacy, and growth environment. For a long time, the method for processing *Coicis Semen* varied, and records of these methods exist in ancient texts, such as The Handbook of Prescriptions for Emergencies (Zhouhou Beiji Fang, Eastern Tsin Dynasty, 341 CE) and the Precious Essential Formulary for Emergency (Beiji Qianjin Yaofang, Tang Dynasty, 652 CE). From the southern and northern dynasties, two processing methods have been described, which involve the frying of glutinous rice and the cooking of salt soup; for example, in the Ming Dynasty, salt frying was employed, whereas in the Qing Dynasty, ginger juice was added to stir-fry preparations. At present, the generally accepted processing methods include stir-fry, soil stir-fry, sand stir-fry, and bran stir-fry techniques, which have been described in various editions of the Chinese Pharmacopoeia since 1985 (Zhou et al., 2020). TCM academic textbooks describe *Coicis Semen* as being effective in relieving dampness and promoting diuresis, strengthening the spleen and preventing diarrhea, eliminating blockages, discharging pus, clearing toxins, and eliminating masses. To date, *Coicis Semen* has been broadly studied in more than 7,500 publications sourced from the Chinese National Knowledge Infrastructure (http://www.cnki.net), the Wanfang Database (https://www.wanfangdata.com.cn/), and the China Science and Technology Journal Database (http://www.cqvip.com/), as well as in approximately 470 articles from the PubMed and Web of Science databases.

Modern phytochemical studies have shown that the botanical drug contains fatty acids and esters, starches and other polysaccharides, flavonoids, triterpenes, alkaloids, sterols, lactams, and other compounds, which exert various therapeutic effects in the treatment of certain diseases (Li X. et al., 2020). Modern pharmacological studies have found that *Coicis Semen* has significant clinical effects, which are mediated through anti-tumor (Manosroi et al., 2019; Huang et al., 2020), anti-inflammatory (Zhang C. et al., 2019), analgesic (Zhang P. et al., 2019), antioxidant (Bai et al., 2019), immune regulatory (Wen et al., 2020), anti-diabetic (Xia et al., 2021), and anti-lipid (Yang Z. et al., 2022) activities, among others (Amen et al., 2017; Chen et al., 2020; Lin et al., 2022).

In recent years, the anti-tumor effects of this botanical drug have been widely investigated, and its efficacy and mechanism of action have been analyzed in-depth through experimental studies and clinical trials. Based on this abundance of information, we aimed to conduct a retrospective analysis of studies involving the clinical application of *Coicis Semen* in the treatment of malignant tumors of the female reproductive system, with a goal of summarizing what is currently known about the toxicity, phytochemistry, pharmacokinetics, anti-tumor effects, and activities leading to reproductive system protection. Ultimately, we summarized the data related to the use of *Coicis Semen* in TCM to promote a better understanding of the clinical mechanisms through which *Coicis Semen* and other drugs can treat female reproductive tumors.

## 2 The antineoplastic activity of *Coicis Semen* in TCM theory

In TCM theory, *Coicis Semen* is a botanical drug with a sweet and light flavor. In TCM, the word “flavor” is not only used to represent the true taste of drugs, but the ancients used flavors to explain their effects as well. For example, *Coicis Semen* has the characteristic of “cold,” which is also an important concept that can represent the properties of botanical drugs and reflect their influence on temperature changes in the human body. In TCM, *Coicis Semen* acts on the spleen, stomach, and lung meridians. The spleen and stomach are the source of the body’s qi and blood, and those zang-fu organs affect the transportation of dampness to the lungs, causing phlegm. The lung is also one of the zang-fu organs that controls the functions and distribution of water and other fluids. The location of the zang-fu organs and meridians mentioned here should not be confused with the anatomical structures in Western medicine; they were first described by ancient physicians who investigated bodily responses after the administration of medications (Zhang et al., 2015). According to the basic properties of TCM, which include four qi and five flavors, the sweet flavor of herbal materials can nourish the qi and blood, and a light flavor is related to the elimination of dampness and diuresis. The essence of water and grain, which the five zang organs and six fu organs require to maintain their normal physiological activities, depends on the movement of the spleen. The intake of *Coicis Semen* can promote healthy transportation and transformation functions of the spleen to ensure the generation of qi and blood is continuous. The cold property is traditionally associated with the elimination or offsetting of excess heat, and *Coicis Semen* can promote the recovery of zang-fu functions and eliminate the accumulation of damp heat in the body. It has often been used to treat diseases such as dysuria, edema, spleen deficiency-related diarrhea, rheumatism, neuralgia, and acute abdominal inflammation (Luo et al., 2018). In TCM, the occurrence of tumors is closely related to the retention of stagnant fluids (Sun et al., 2021); this also affects the smoothness of blood vessels, which, in turn, leads to phlegm turbidity and static blood accumulation. These changes can cause masses to form over time and are accompanied by heat toxins; when these pathological products coalesce in the female reproductive organs, gynecological malignancies are induced. The theory of “Yin and Yang” is based on the maintenance of a dynamic equilibrium, which is the cornerstone for understanding the ways in which the human body interacts with all things in TCM. A deficiency of healthy qi and the blockages caused by pathological processes, including dampness, phlegm accumulation, and blood stasis will lead to imbalances within the human body. The destruction of the original equilibrium between Yin and Yang creates a suitable environment for tumor growth and metastasis. Treatment with *Coicis Semen* can restore the body’s ability to maintain the dynamic balance between Yin and Yang and improve qi–blood circulation through heat clearance, alleviation of dampness, and the removal of toxins; these changes promote the dispersion of nodules and result in antineoplastic activity.

## 3 *Coicis Semen* in TCM clinical trials for treating malignant tumors of the female reproductive system

The literature search ultimately led to the identification of 74 clinical trials on the efficacy of herbal prescriptions containing *Coicis Semen* for the treatment of female reproductive cancers, including, but not limited to, cervical cancer and ovarian cancer, and complications related to the disease or treatment. *Coicis Semen* is widely used in the treatment of malignant tumors of the female reproductive system in combination with other TCM such as *Atractylodis Macrocephalae Rhizoma, Astragali Radix, Poria, Glycyrrhizae Radix et Rhizoma*, and *Hedyotis diffusae Herba* (Supplementary Table S1; Table 1). Some related studies have been reported in some researchers’ reviews and medical records.

**TABLE 1:** The number of studies utilizing prescriptions containing *Coicis Semen* for the management of malignant tumors of the female reproductive system.

Accepted medicinal plant name	Latin name of herb	Frequency
*Atractylodes macrocephala* Koidz.	*Atractylodis Macrocephalae Rhizoma*	52
*Astragalus membranaceus* (Fisch.) Bunge	*Astragali Radix*	49
*Poria cocos* (Schw.) Wolf	*Poria*	42
*Glycyrrhiza uralensis* Fisch.	*Glycyrrhizae Radix et Rhizoma*	38
*Hedyotis diffusa* Willd.	*Hedyotis Diffusae Herba*	26
*Dioscorea opposita* Thunb.	*Dioscoreae Rhizoma*	26
*Codonopsis pilosula* (Franch.) Nannf.	*Codonopsis Radix*	23
*Angelica sinensis* (Oliv.) Diels	*Angelicae Sinensis Radix*	22
*Scutellaria barbata* D.Don	*Scutellariae Barbatae Herba*	21
*Citrus reticulata* Blanco	*Citri Reticulatae Pericarpium*	20

A total of 3,113 patients were included in the 74 studies. Because of the complexity and refractory treatment of malignant tumors, in the 74 clinical trials, the majority of studies (55 studies) aimed to evaluate the advantages of combining TCM and Western medicine-based therapies (e.g., radiotherapy, chemotherapy) compared with the effects of treatment with Western medicine alone, and the herbal formulations administered contained between four and 26 TCM ingredients (most commonly 10–20). Most studies have focused on the treatment of cervical cancer and ovarian cancer, mainly by analyzing the treatments’ overall effects on the tumor. Some of the studies focused on the treatment or prevention of malignant ascites and radiotherapy-induced adverse reactions, such as radiation enteritis. The overall efficacy of the treatments for malignant tumors varied between 51.2% and 96.43%, the efficacy of treatments targeted at ascites was between 79.5% and 88.1%, and the efficacy of treatments for radiation enteritis ranged from 86% to 97.62%; these effects were better than those seen in the control groups (the therapeutic ranges in the control groups were 27.30%–87.5%, 42.86%–80.5%, and 40%–78.26%, respectively) and are summarized in Supplementary Table S1. The evidence from these clinical trials demonstrates that *Coicis Semen* may play a role in enhancing the short-term efficacy of the anti-tumor treatments, improving the quality of life of patients, and reducing the side effects of radiotherapy and chemotherapy. In the treatment of malignant tumors of the female reproductive system, the combination of TCM and Western medicine was more beneficial than Western medicine alone.

The inclusion of a placebo-treated control group is important for ensuring the scientific rigor of clinical trials. In rigorous double-blinded clinical trials, proper blinding can be achieved when the test drug and placebo are consistent in terms of their appearance, smell, packaging and labels, and other characteristics. However, due to the complexity of the components in TCM preparations, it can be very difficult to generate a placebo formulation that cannot be differentiated from the TCM. In particular, many TCM preparations investigated in clinical trials are very distinctive in terms of their characteristic odor. Therefore, even if a placebo is used, there is a possibility that the control and treatment groups may be identifiable. In addition, due to the particularities of clinical trials in oncology, it is often difficult to achieve proper blinding in those involving TCM. Among the 74 studies, 66 were randomized, controlled trials without blinding (open trials), and seven were non-randomized, controlled trials. The one remaining trial conducted statistical analyses comparing the data before and after treatment within subjects. Few of the clinical trials were randomized, double-blinded, placebo-controlled, multicentered studies.

The clinical trials that investigated the treatment of malignant gynecological tumors with formulations containing *Coicis Semen* were characterized by small sample sizes (most commonly fewer than 70 patients/group) and a relatively short duration of treatment (mostly 4 weeks to 6 months). In terms of indicators of therapeutic outcomes, the endpoints for the short-term efficacy of the treatments were based on tumor responses, changes in tumor markers or immune function, adverse reactions, the Karnofsky Performance Status (KPS), and the TCM syndrome score. An overall assessment of these studies is difficult because of differences in the duration of the clinical trials, the geographic location and ethnicity of patients, and the quality control processes related to the administration of TCM (Liu et al., 2016; Qu et al., 2019). In addition, prolongation of survival is an important therapeutic concern for patients with cancer; however, only five clinical trials have followed up on survival times. In the future, it will be necessary to further improve the design of such trials, to assess a greater number of survival indicators such as overall survival (OS) and progression-free survival (PFS), and to provide more convincing clinical evidence.

In the trials on the treatment of malignant tumors of the female reproductive system, 4–26 TCM ingredients were involved. It is worth noting that the recommended dose of *Coicis Semen* mentioned in the Chinese Pharmacopoeia (2020 version) is 9–30 g, and the TCM prescriptions included in the studies mostly conformed to this criterion, with relative weight contributions varying from 4.44% to 36.36%, as listed in Supplementary Table S1. Factors such as the picking season and origin of the botanical drugs, the processing technology used, and individual differences in patient characteristics can affect the efficacy of TCM formulations and their associated adverse reactions, and it will be important to control for these factors in future trials (Sang et al., 2018; Zhang et al., 2022). In addition, TCM can comprehensively regulate bodily processes by mediating a variety of physiological transformations and interactions, offering a holistic approach to the overall modulation of signal transmission networks and responses based on herbal compatibility. Among the included studies, few discussed the interaction between different Chinese medicines and the potential toxicity risk. Therefore, in future clinical trials, individual compounds in Chinese herbal prescriptions should be studied separately, and researchers should aim to strengthen the interaction between TCM and Western medicine-based approaches to promote the rational use of TCM, prolong survival times, and improve the quality of life of patients.

## 4 Phytochemistry of *Coicis Semen*

**FIGURE 2:**
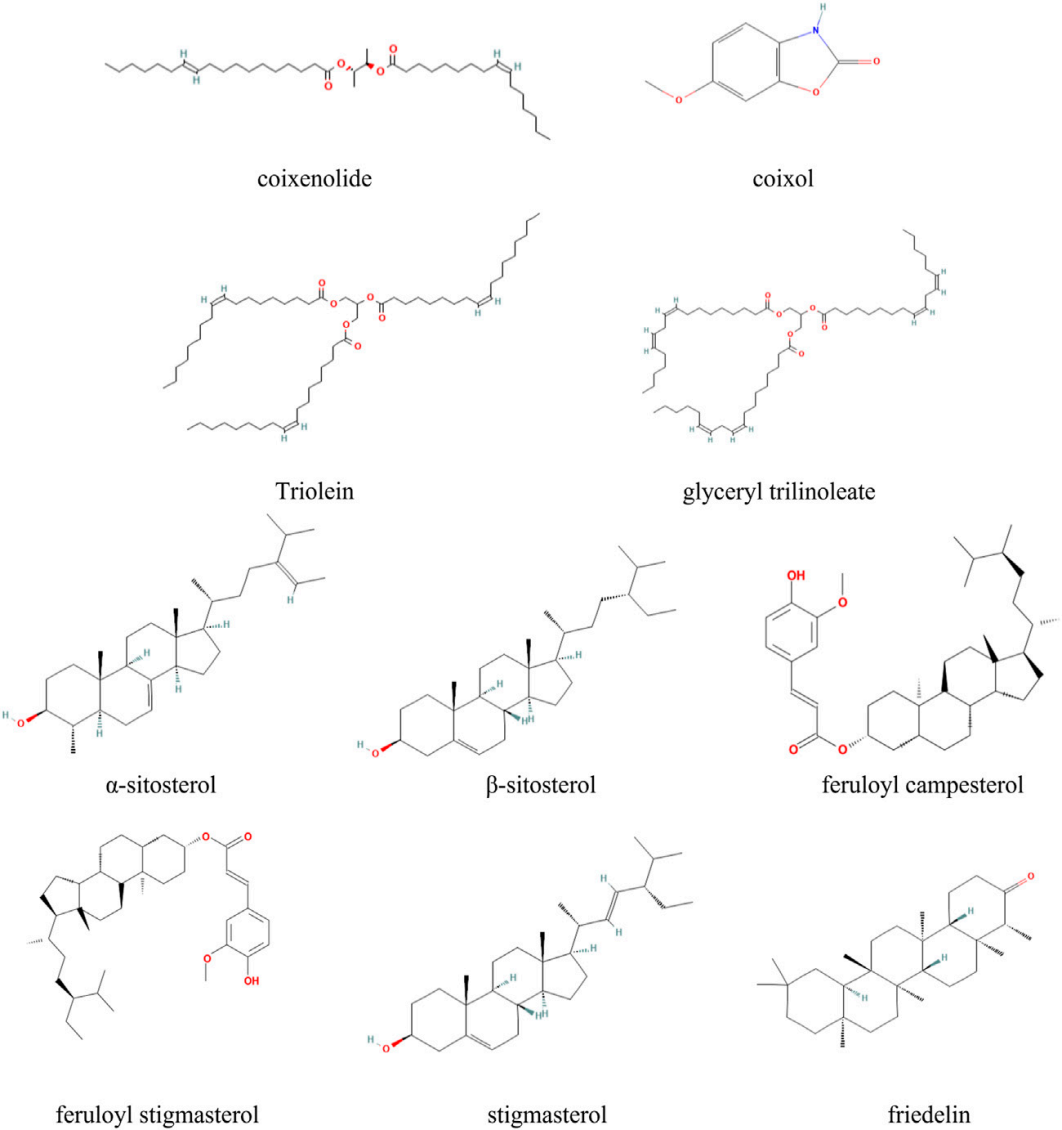
Structures of the major active compounds present in *Coicis Semen.*

At present, more than 80 compounds have been isolated from *Coicis Semen*, including mainly starches and other polysaccharides, fatty acids and esters, proteins, and various nutrients such as phenolic acids, sterols, flavonoids, lactams, triterpenes, alkaloids, and adenosine, with unsaturated fatty acids, esters, polysaccharides, and triterpenoids being the main active components; some of these compounds are listed in Table 2, and the structures of the major active compounds present in *Coicis Semen* are depictured in Figure 2. The extraction of various compounds from *Coicis Semen* laid the foundation for the in-depth study of the pharmacological mechanism through which the botanical drug exerts anti-tumor effects *via* multiple targets and pathways.

**TABLE 2:** Constituent ingredients of *Coicis Semen*, compiled from the HERB database (http://herb.ac.cn).

Ingredient ID	Ingredient name	Molecular formula	Ingredient ID	Ingredient name	Molecular formula
HBIN000890	1,2-linoleic acid-3oleic acid- triglyceride	C_57_H_100_O_6_	HBIN005609	2-ethyl-3-hydroxyhexyl butyrate	C_12_H_24_O_3_
HBIN006106	2-monoolein	C_21_H_40_O_4_	HBIN006366	[(2R)-2,3-dihydroxypropyl] (Z)-octadec-9-enoate	C_21_H_40_O_4_
HBIN012837	(6Z,10E,14E,18E)-2,6,10,15,19,23-hexamethyltetracosa-2,6,10,14,18,22-hexaene	C_30_H_50_	HBIN015611	α-monolinolein	C_21_H_38_O_4_
HBIN015675	α-sitosterol	C_30_H_50_O	HBIN016562	arabinose	C_5_H_10_O_5_
HBIN016720	Arginine	C_6_H_14_N_4_O_2_	HBIN018278	beta-sitosterol	C_29_H_50_O
HBIN019257	Cadmium	Cd	HBIN019351	calcium	Ca
HBIN019475	Campesterol	C_28_H_48_O	HBIN019688	caprylic acid	C_8_H_16_O_2_
HBIN021150	Cholesterol	C_27_H_46_O	HBIN021250	coixan A	——
HBIN021251	coixan B	——	HBIN021252	coixan C	——
HBIN021253	Coixendide	C_38_H_70_O_4_	HBIN021254	coixenolide	C_38_H_70_O_4_
HBIN021256	Coixol	C_8_H_7_NO_3_	HBIN021258	colchamine	C_21_H_25_NO_5_
HBIN021426	Copper	Cu	HBIN024930	linoleic acid	C_18_H_32_O_2_
HBIN025553	Ergosterol	C_28_H_44_O	HBIN025965	ethylpalmitate	C_18_H_36_O_2_
HBIN026471	feruloyl ampeaterol	C_38_H_58_O_4_	HBIN026474	feruloyl campesterol	C_38_H_58_O_4_
HBIN026477	feruloyl stigmasterol	C_39_H_60_O_4_	HBIN026766	friedelin	C_20_H_18_O_5_
HBIN026982	gaidic acid	C_16_H_30_O_2_	HBIN026990	galactose	C_6_H_12_O_6_
HBIN027997	glucan1	——	HBIN027998	glucan2	——
HBIN027999	glucan3	——	HBIN028000	glucan4	——
HBIN028001	glucan5	——	HBIN028002	glucan6	——
HBIN028003	glucan7	——	HBIN028037	glucose	C_6_H_12_O_6_
HBIN028100	Glyceride	C_16_H_32_O_4_	HBIN028131	glyceryl trilinoleate	C_57_H_98_O_6_
HBIN029313	Hexanal	C_6_H_12_O	HBIN029328	hexanoic acid	C_6_H_12_O_2_
HBIN030085	Indene	C_9_H_8_	HBIN030436	isoarborinol	C_30_H_50_O
HBIN032825	Lead	Pb	HBIN032990	leucine	C_6_H_13_NO_2_
HBIN033339	linolenic acid	C_18_H_30_O_2_	HBIN034051	lysine	C_6_H_14_N_2_O_2_
HBIN034204	Magnesium	Mg	HBIN034389	mandenol	C_20_H_36_O_2_
HBIN034390	Manganese	Mn	HBIN034421	mannose	C_6_H_12_O_6_
HBIN034592	6-Methoxy-2-benzoxazolinone	C_8_H_7_NO_3_	HBIN034760	mercury	Hg
HBIN035296	methyl linoleate	C_19_H_34_O_2_	HBIN035345	methyl oleate	C_19_H_36_O_2_
HBIN036159	myristic acid	C_14_H_28_O_2_	HBIN037254	non-anoic acid	C_9_H_18_O_2_
HBIN037732	octadecadienoic acid	C_18_H_32_O_2_	HBIN037764	octadecenoic acid	C_18_H_34_O_2_
HBIN038026	oleic acid	C_18_H_34_O_2_	HBIN038034	olein	C_57_H_104_O_6_
HBIN038088	Omaine	C_21_H_25_NO_5_	HBIN038680	palmitic acid	C_16_H_32_O_2_
HBIN039626	Phosphorus	P	HBIN042172	rhamnose	C_6_H_12_O_5_
HBIN042712	(S)-4-Nonanolide	C_9_H_16_O_2_	HBIN044158	sitosterol	C_29_H_50_O
HBIN044161	alpha1-sitosterol	C_30_H_50_O	HBIN044730	stearic acid	C_18_H_36_O_2_
HBIN044918	Stigmasterol	C_29_H_48_O	HBIN046757	trans-feruloylsigmastenol	C_39_H_60_O_4_
HBIN046760	trans-feruloylcampesterol	C_38_H_58_O_4_	HBIN047094	trilinolein standard	C_57_H_98_O_6_
HBIN047137	Triolein	C_57_H_104_O_6_	HBIN047720	valine	C_5_H_11_NO_2_
HBIN047744	Vanillin	C_8_H_8_O_3_	HBIN048918	zinc	Zn

Coix seed oil, as an effective extract of *Coicis Semen*, is mainly composed of neutral oil, including ester compounds and fatty acids. Coix seed neutral oil predominantly comprises triglycerides (85%), followed by monoglycerides, diglycerides, and fatty acyl hydrocarbon esters (Xiang et al., 2005; Hou et al., 2018). Due to the different types, proportions, and arrangements of fatty acids, the composition of synthesized triglycerides varies widely, as do the needs of the human body, with fatty acid residues comprising hexadecanoic acid, octadecanoic acid, octadeconenoic acid, and octadecadienoic acid. Of these components, triglycerides (including but not limited to triolein, glyceryl trilinoleate, and 1,2-linoleic acid-3-oleic acid triglyceride) mainly play an anti-tumor role and were described as an indicator for evaluating the quality of *Coicis Semen* preparations in the 2010 edition of the Pharmacopeia of the People’s Republic of China. Coix seed oil also contains coixenolide, coixol, and other important components. As early as 1961, the Japanese scholar Ukita and his colleagues first isolated coix seed ester from *Coicis Semen* and provided evidence of its anti-tumor effect (Ukita & Tanimura, 1961). Because this ingredient is a natural chemical product and is associated with few adverse reactions, its pharmacological effects have attracted much attention since its initial discovery. Studies have shown that the injection of Kanglaite, a patented Chinese medicine developed from Coix seed oil as the raw material, induces a good therapeutic response in the treatment of gastric, colorectal, and lung cancers, among others. Some studies have also discussed its mechanism of action, demonstrating that the fatty acids and esters in *Coicis Semen* can exert anti-tumor effects by inhibiting the proliferation and metastasis of tumor cells (Xiong et al., 2018; Lin et al., 2019; Ni et al., 2021), preventing tumor angiogenesis (Zhang, 2020), increasing the sensitivity to radiotherapy (Liu J. et al., 2018), and improving the body’s immunity (Zhang, 2017).

*Coicis Semen* is also rich in polysaccharides, including coixan A, B, and C, acidic polysaccharides CA-1 and CA-2, neutral glucans 1–7, and fructo-oligosaccharides (Chen et al., 2019; Hu et al., 2022). The monosaccharides constituting these polysaccharides mainly include mannose, rhamnose, glucose, and arabinose. A large number of studies have shown that the polysaccharides exert anti-oxidative effects (Li et al., 2012), while regulating blood glucose levels (Chen et al., 2019) and immune functions (Lv et al., 2013; Wang et al., 2021). In addition, it has been reported that the polysaccharides extracted from *Coicis Semen* can also directly induce tumor cell apoptosis (Lu et al., 2013), and the polysaccharides can also act synergistically in combination with chemotherapeutic agents in cancer treatment (Pang et al., 2018). Its composition results in strong stability, and it is associated with few adverse reactions. Not only can it destroy tumor cells directly, but it can also activate immune cells to indirectly attack tumors, and its immune regulatory effects are evident, even at relatively low concentrations. Experimental studies have found that coixan can inhibit the progression of liver cancer and lung cancer (Liu et al., 2015; Luo et al., 2017). Based on the characteristics of the active ingredients contained in TCM preparations that exert anti-tumor effects, some studies have also designed and constructed appropriate nanoparticle or microparticle drug delivery systems by regulating the particle size, surface properties, drug carrier type, and other technical properties; such delivery systems could be used to overcome the low oral bioavailability of the polysaccharides contained in *Coicis Semen*, thereby enhancing the anti-tumor efficacy (Yuan et al., 2014; Liu et al., 2015; Qu et al., 2016).

*Coicis Semen* also contains a variety of sterols, such as α-sitosterol, β-sitosterol, feruloyl campesterol, feruloyl stigmasterol, and stigmasterol. Some studies have shown that sterols can inhibit the proliferation, cell cycle progression, and aggregation of malignant tumor cells (Baskar et al., 2012; Blanco-Vaca et al., 2019; Bae et al., 2021). Two types of triterpenoids have been isolated from *Coicis Semen*—friedelin and cylindrin—and these compounds have been proven to exert anti-cancer (Li S. et al., 2020; El-Baba et al., 2021), antiviral (Wu et al., 2020), hypoglycemic (Bian et al., 2021), and hypolipidemic (Liu Y. et al., 2018) effects.

Different processing methods have a certain influence on the chemical composition of *Coicis Semen*. Studies have found a higher content of free fatty acids, amino acids, triterpenes, and phenols in fermented *Coicis Semen*, which is helpful for regulating metabolism and lowering blood pressure. Fermentation also reduced the amount of the hazardous substance 2-pentylfuran and improved the safety profile of *Coicis Semen* (Yin et al., 2020). The content of free phenolic acid was also shown to increase in germinated *Coicis Semen*, and the antioxidant activity was significantly improved, with its extract being capable of inhibiting the proliferation of human colon cancer cells and inducing the apoptosis of cervical cancer cells (Son et al., 2017; Xu et al., 2017). By comparison, it was found that the triglyceride content in *Coicis Semen* products generated through different processing techniques was higher than that of other products (raw product (soil-fried product (0.7618%) > pure fried product (0.7016%) > bran-fried product (0.5682%) > raw product (0.5442%)) (Shen et al., 2015). The experimental results of one study also showed that fried Coix seed oil can promote intracellular oxidative stress and inhibit breast cancer (Zheng et al., 2020).

## 5 The pharmacokinetics of *Coicis Semen*

In pharmacokinetic experiments, researchers have used tritium labeling to dynamically observe the absorption, distribution, and metabolism of Coix seed oil preparations in rats after administration *via* gavage or tail vein injection. The elimination half-life of the intravenous preparation was 15.84 h, whereas that of the oral preparation was 14.23 h, suggesting slow drug metabolism and delayed excretion. At the same time, comparisons of the area under the curve (AUC) revealed that the bioavailability of oral preparations was equivalent to 62% of that of intravenous preparations. After intravenous injection of a Coix seed oil preparation, the drug was widely distributed in various tissues and organs, with the highest concentrations observed in the liver and spleen. The total proportion of the drug excreted in 24 h was 38.29%, 59.4% and 40.6% of which occurred through urine and feces, respectively. The plasma protein binding rate of Coix seed oil was 98.4% *in vitro* and 80.5% *in vivo* (Li et al., 2005).

Another study determined the content of olein in rat plasma by ultra-high-performance liquid chromatography–mass spectrometry (UPLC-MS). After administration of Coix seed oil raw material, Kanglaite soft capsules, and nanoparticles of Coix seed oil, the ρ_max_ values of plasma olein in rats were 5.43 ± 0.45 mg/L, 7.54 ± 0. 44 mg/L, and 7.30 ± 1.13 mg/L, respectively. Compared with that of the raw material, the ρ_max_ of olein after the administration of nanoparticles was significantly higher. The AUC_0-∞_ values of plasma olein in rats were 68.71 ± 5.12 mg/h/L, 46.61 ± 3.86 mg/h/L, and 178.91 ± 6.26 mg/h/L for the three preparations, respectively, with the nanoparticles having the highest values. The relative bioavailability of olein for the nanoparticle preparation was 260.38%, with Coix seed oil raw material used as a reference. When using the common oral preparation of Coix seed oil as a reference, the relative bioavailability of olein in the nanoparticle preparation was 383.84%, suggesting that the nanoparticle system could increase the oral bioavailability of Coix seed oil and enhance its pharmacological properties (Huang Q. et al., 2016).

In terms of drug interactions, one *in vivo* study investigated the effect of Coix seed oil on cytochrome P450 (CYP) enzymes (CYP1A2, CYP2B6, CYP2C9, CYP2C19, and CYP3A4) by using cocktails of probe drugs in rats. The rats were administered a single oral dose (2.5 mL/kg body weight) of Coix seed oil 1 h before administration of a drug cocktail, either orally or intravenously, and blood was collected at various time points. The results showed that a single oral dose of Coix seed oil did not affect the pharmacokinetics of the five probe drugs when given as a drug cocktail intravenously; however, when Coix seed oil was administered concomitantly with other drugs, a single oral dose or short-term dosing could increase plasma drug concentrations and enhance intestinal absorption (Yao et al., 2019). Another study also looked at the effect of Kanglaite on the expression of rat CYP enzymes and showed that treatment with multiple doses of Kanglaite (for 7 days *via* intraperitoneal injection) had an induction effect on rat CYP1A2, whereas CYP2B6, CYP2C9, CYP2C19, and CYP3A4 enzyme activities were inhibited (Du et al., 2015). Therefore, it is necessary to take precautions to avoid possible interactions between Coix seed oil and other drugs in future applications.

At present, there are few studies on the pharmacokinetics of *Coicis Semen*; those that do exist have all focused on Coix seed oil, which has proven anti-tumor effects. However, the effects of other active ingredients, such as coixan polysaccharides and triterpenoids, on tumors remain unknown. Therefore, future pharmacokinetic studies must focus on improving the sensitivity of the analytical methods to facilitate a deeper analysis of a series of metabolic processes, including the absorption, distribution, and excretion of active ingredients in *Coicis Semen* preparations.

## 6 Anti-*tumor* effects and protection of reproductive function by *Coicis Semen* and its constituents in the treatment of gynecologic malignant tumors

**FIGURE 3:**
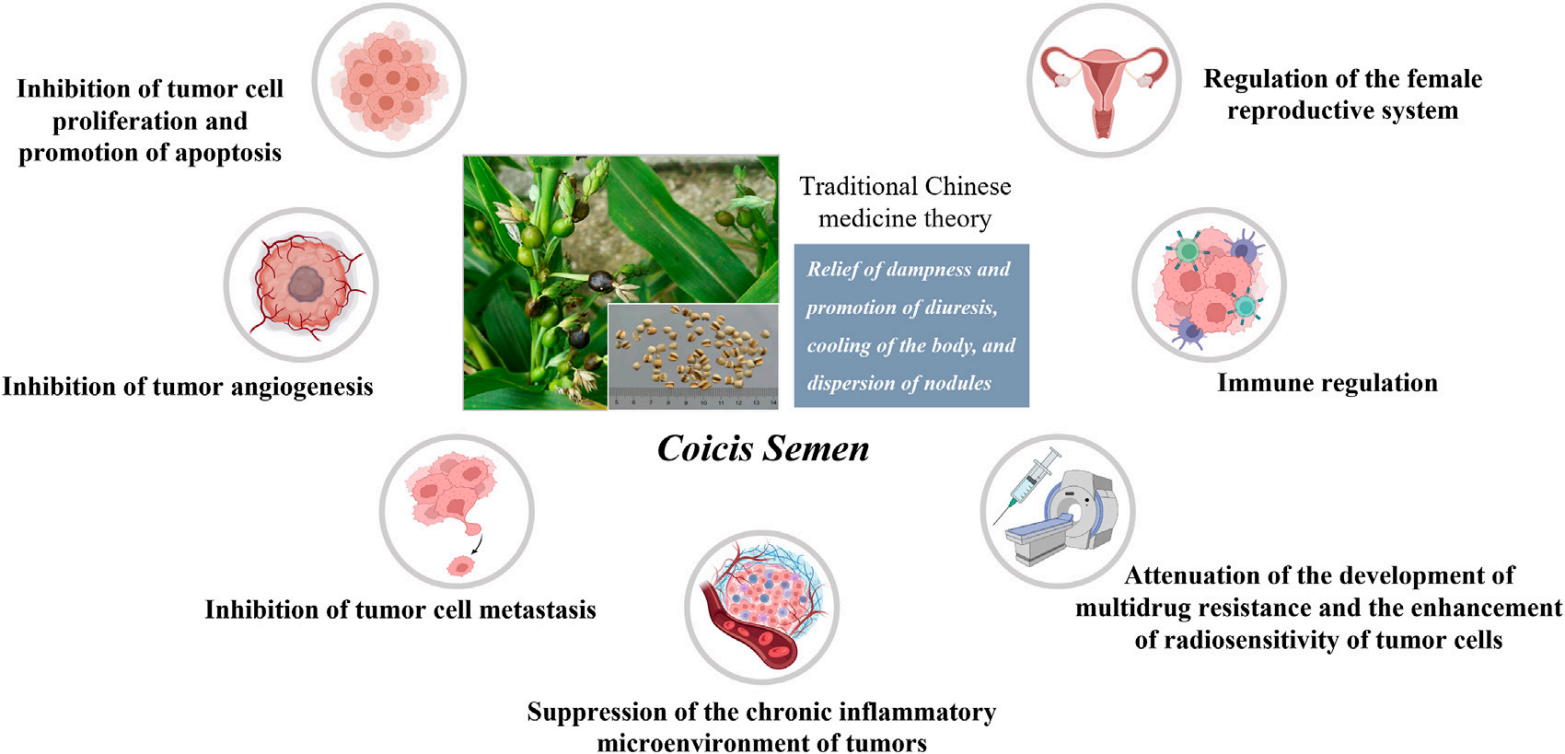
Anti-tumor effects and protection of reproductive function by *Coicis Semen*.

A number of *in vitro* and *in vivo* studies have reported beneficial effects of *Coicis Semen* and its constituents in the treatment of malignant tumors of the female reproductive system, including cervical cancer (Guo et al., 2019), ovarian cancer (Xu et al., 2021), endometrial cancer (Huang et al., 2021a), and uterine sarcoma (Chang et al., 2018). In this section, we summarize the possible mechanisms through which *Coicis Semen* and its related active components exert anti-cancer effects and regulate reproductive function in order to provide support for the possible use of this botanical drug in the treatment of gynecologic malignant tumors (Figure 3).

### 6.1 Inhibition of tumor cell proliferation and promotion of apoptosis

Adlay (*Coix lachryma-jobi L.*) is an annual crop. Researchers have demonstrated that the hexane fraction of adlay testa ethanolic extracts (ATE-Hex) inhibited the proliferation of the human sarcoma cell lines MES-SA and MES-SA/Dx5, and treatment with ATE-Hex alone or in combination with doxorubicin significantly inhibited the growth of uterine sarcoma cells and induced apoptosis by increasing the number of cells in the sub-G1 phase as well as the amount of poly (ADP-ribose) polymerase (PARP) being cleaved (Chang et al., 2018). Huang et al. (2021a) found that endometrial cancer cell growth and cell cycle arrest occurred most significantly in the G1 or G2/M phase in response to treatment with the ethyl acetate fraction of the adlay testa ethanolic extract (ATE-EA) ranging from 25 to 200 μg/mL for 48 h. Another study also confirmed that the fractions and subfractions of adlay seed ethanolic extract (starting from 12.5 μg/mL for 72 h) inhibited Hela cell proliferation, induced cell cycle arrest in the G0/G1 phase, and decreased cyclin-dependent kinase 4 (CDK4)/cyclin D1 protein expression (Chiang et al., 2022). Furthermore, *Coicis Semen* and its active components can also exert pharmacological effects that involve the inhibition of cell proliferation and the mediation of apoptosis in lung (Chang et al., 2003), breast (Fang et al., 2020), pancreatic (Yang J. et al., 2022), colon (Lee et al., 2008), and other cancers.

### 6.2 Inhibition of tumor angiogenesis

Tumor blood vessels are nutrient channels and metastatic pathways for carcinoma cells. In general, if angiogenesis is inhibited or blood flow is blocked, cells will be deprived of oxygen and energy, which can inhibit the growth of carcinoma cells. In an experimental model, the antitumor activity of icaritin (IC) and Coix seed oil dual-loaded multicomponent thermosensitive lipid complexes (IC-ML) was evaluated in BALB/c nude mice bearing desmoplastic tumors under mild hyperthermic conditions. Compared with that of the IC group, the tumor size and growth curve were effectively inhibited in groups treated with IC and Coix seed oil co-loaded microemulsions (IC-MEs), including the IC-ML(H+) and IC-ML(H-) groups, in which the IC-ML preparations were incubated at higher and lower temperatures of 42°C and 37°C, respectively. In the subsequent western blotting experiment, it was confirmed that IC-ML(H+) could inhibit tumor angiogenesis and promote blood vessel restoration through deactivation of the hypoxia-inducible factor 1 alpha–vascular endothelial growth factor (HIF-1α-VEGF) pathway (Guo et al., 2021). Other studies have also shown through *in vivo* and *in vitro* experiments that the active components in *Coicis Semen* exert a significant inhibitory effect on tumor angiogenesis, and reducing the expression of VEGF, basic fibroblast growth factor (bFGF), and VEGF receptor-2/kinase-insert domain-containing receptor (VEGFR-2/KDR) proteins may be the intrinsic mechanism of action (Feng et al., 2004; Shen et al., 2013), with the dose range of *Coix Lacryma-jobi* injection is 6.25 mL/kg–25 mL/kg. Several studies have confirmed that angiogenesis plays a key role in the occurrence and development of various female reproductive system malignancies. Therefore, based on the effect of *Coicis Semen* on tumor blood vessel formation, it is possible for this TCM to treat female reproductive system tumors by inhibiting tumor angiogenesis and reducing tumor blood supply.

### 6.3 Inhibition of tumor cell metastasis

Luo et al. (2017) found that the polysaccharide fraction extract CP1 inhibited the migration and invasion of A549 cells (a human non-small cell lung cancer cell line) by downregulating the gene and protein expression levels of S100 calcium binding protein A4 (S100A4), with possible interaction with the binding site of S100A4–non-muscle myosin IIA (NMIIA). Son et al. (2017) confirmed through an *in vitro* experiment that treatment with 1 mg/mL *Coix lacryma-jobi* var. *ma-yuen* Stapf sprout extract (CLSE) resulted in a 54% reduction in the hypoxia-induced invasiveness of colon cancer cells and a 50% inhibition of adhesive potency through inactivation of the extracellular signal-regulated kinase (ERK) 1/2 and protein kinase b (AKT) pathways. There was also evidence that Coix seed oil could interfere with the invasion and migration ability of various tumor cells by regulating the expression of the enzyme protein arginine methyltransferase 5 (PEMT5) and migration-induced gene 7 (MIG-7), thereby downregulating the expression levels of platelet-derived growth factor and its receptor and inhibiting the activity of the phosphoinositide 3-kinase/Akt (PI3K/Akt) axis (Yu et al., 2016; Sun et al., 2019; Fan and Zhao, 2022). Similar to that of other malignancies, cancer cell metastasis in patients with malignant tumors of the female reproductive system is also the main factor that impacts the curative effect of the treatment and the resulting poor prognosis. Thus, *Coicis Semen* can play a therapeutic role by inhibiting the invasion and migration of tumor cells.

### 6.4 Suppression of the chronic inflammatory microenvironment of tumors

Inflammation is a means of self-protection in the body that is triggered by the host’s system in response to pathogens, leading to the rapid activation of the innate immune response. In general, inflammation is beneficial as it promotes healing; however, if inflammation is excessive or becomes chronic and persistent, it can have deleterious effects. Chen et al. (2021) found that Kanglaite injection (2 mg/mL) for 24 h could reduce inflammation of the tumor microenvironment by inhibiting the chemokine-like factor 1 (CKLF1)-mediated nuclear factor kappa B (NF-κB) pathway. In addition, Kanglaite injection can reduce the expression level of NF-κB in the nuclei of lung cancer cells in a dose-dependent manner (at a dose of 6.25 or 12.5 mL/kg), as well as the expression levels of the inhibitor of NF-κB alpha (IκBα), IκB kinase (IKK), and epidermal growth factor receptor (EGFR) (Pan et al., 2012). *Coicis Semen* extract (100 and 200 mg/kg) can also exert anti-inflammatory effects by reducing the levels of the pro-inflammatory cytokines interleukin 1 beta (IL-1β), tumor necrosis factor alpha (TNF-α), interleukin 6 (IL-6), and monocyte chemoattractant protein 1 (MCP-1) (Zhang C. et al., 2019). *Coicis Semen* extract (1, 2.5 and 5 mg/mL) can exert anti-tumor effects in MDA-MB-231 breast cancer cells by downregulating the expression of NF-κB, protein kinase C, cyclooxygenase 2 (COX-2), and matrix metalloproteinases (Woo et al., 2007). The ethanolic extract of *Coicis Semen* exhibits potent anti-inflammatory activity by inhibiting COX-2 expression in RAW264.7 macrophages and lung cancer cells (Hung and Chang, 2003; Huang et al., 2009). The occurrence of cervical cancer (Nahand et al., 2020), ovarian cancer (Macciò and Madeddu, 2012), and endometrial cancer (Alper et al., 2021) is closely related to inflammation; therefore, we believe that *Coicis Semen* can exert its therapeutic potential by modulating these inflammatory pathways.

### 6.5 Attenuation of the development of multidrug resistance (MDR) and the enhancement of radiosensitivity of tumor cells

The success of pharmacological treatment of many malignant tumors can be negatively impacted by the development of MDR. As the number of treatment cycles increases, drug sensitivity gradually decreases, and chemotherapeutic resistance seriously affects drug efficacy. Many studies have found that the active components present in *Coicis Semen* can reduce drug resistance, and to date, many of those that have investigated the pathophysiological mechanisms driving MDR have focused on the decrease in drug uptake and the increase in efflux transporters in tumor cells (Auner et al., 2010; Khan et al., 2017). The involvement of members of the ATP-binding cassette (ABC) transporter family is closely related to the development of MDR (Assaraf et al., 2019). A number of studies have shown that the administration of *Coicis Semen* extract can reverse the development of MDR, enhance the cellular sensitivity to chemotherapeutic drugs, and significantly halt the growth of tumor cells by inhibiting the efflux function and downregulating the expression levels of ABC transporters, such as P-glycoprotein, multidrug resistance-associated protein 2, and breast cancer resistance protein (Wang et al., 2017; Zhang et al., 2017; Zhu et al., 2018; Qian et al., 2019; Chen et al., 2021). In addition to helping minimize resistance to chemotherapy, *Coicis Semen* can be used as an adjuvant therapeutic agent during radiotherapy. For example, Hu et al. (2000) used ^60^Co as a radioactive source and demonstrated, using microcolony formation assays, that coixenolide (10^−7^–10^–6^ mol/L) can enhance the sensitivity of CNE-2Z cells to gamma rays.

### 6.6 Immune regulation

The occurrence of tumors is largely due to a decline in the body’s immune function and the inability to recognize “other” cells, resulting in the infinite proliferation of tumor cells. Lymphocytes and innate immune cells protect colonic tissue by regulating the inflammatory response in ulcerative colitis, and studies have found that mice with colitis who are fed a diet containing *Coicis Semen* could experience a change in colonic T lymphocyte subsets and innate immune cell abundance (Zhou et al., 2021). Furthermore, NF-κB can promote cell survival and stimulate immune responses, while Kanglaite injection (6.25 and 12.5 mg/kg) can exert anti-cancer and immunomodulatory effects and regulate immune activity by inducing NF-κB-mediated gene transcription in CD4^+^ T cells (Huang et al., 2014), and it can also activate the immunogenicity of Lewis lung cancer cells (Huang et al., 2009). Natural killer (NK) cells are not only related to anti-tumor and antiviral responses and immune regulation, but they are also involved in the occurrence of hypersensitivity and autoimmune diseases in some cases, and they can recognize target cells and act as mediators of their destruction. There are many surface markers expressed by NK cells, including cluster of differentiation (CD) complexes CD3, CD56, CD16, and CD57, among others. Hidaka et al. (1992) administered six capsules of *Coicis Semen* to seven volunteers three times a day for four consecutive weeks (110 mg of dried extract of Coix seeds in one tablet) and observed a significant increase in the percentage of CD3^+^/CD56^+^ cells (major histocompatibility complex (MHC)-non restricted cytotoxic T cells) and CD16^+^/CD57^-^ cells (the mature and most active NK cells). The levels of CD3^-^/CD56^+^ (NK cells) and CD16^+^/CD57^+^ (variably active NK cells) decreased at 1 week and returned to normal levels thereafter, which is basically consistent with the experimental results reported by Kaneda et al. (1992).

### 6.7 Regulation of the female reproductive system

The occurrence and development of gynecologic tumors are closely related to the levels of female sex hormones. In a mouse model expressing the human papillomavirus (HPV) oncogene, exogenous estrogen administration promotes cervical cancer through stromal estrogen receptor alpha. In contrast, high estradiol and low progesterone levels increase cervical cancer survival (Hellberg, 2012; Lee et al., 2021). In ovarian cancer, steroid hormones, gonadotropins, estrogens, and androgens promote its progression, whereas gonadotropin-releasing hormone and progesterone inhibit it (Li et al., 2021). In addition, insufficient progesterone activity significantly increases the risk of endometrial cancer (Kim et al., 2013). Therefore, the regulation of female reproductive hormones is an important means of treating gynecologic malignant tumors. Lin et al. (2019) and the experimental studies of others found that *Coicis Semen* can attenuate the uterine hyperplasia induced by diethylstilbestrol/medroxyprogesterone 17-acetate (DES/MPA) and inhibit the proliferation of human uterine leiomyoma. Hsia et al. (2007) found that *Coicis Semen* could reduce the secretion of progesterone and estradiol in rats. Finally, Huang et al. (2021b) and other experimental studies showed that the ethanol extract from *Coicis Semen* shells administered at a dose of 30 mg/day could relieve the contraction of myometrial tissue caused by oxytocin and acetic acid.

## 7 Side-effects and toxicity

As a common food product and homologous TCM, *Coicis Semen* has remarkable medicinal effects, although there are few reports on its toxicity and adverse reactions. Tao et al. (2013) conducted tests on the acute toxicity as well as the skin and rectal irritation induced by Coix seed oil; the study showed no obvious acute toxicity in mice and suggested it is safe for oral and external use. After administering the soft capsule contents of Coix seed oil [17.4 g/(kg·day)] to mice, no abnormalities were observed in terms of their general condition, food utilization rate, body weight, organ weight, organ ratio, or routine blood and biochemical indexes (Huang, 2020). Another two studies (Fan et al., 2000; Xiao, 2009) confirmed that Coix seed oil and coixan polysaccharides were safe and did not acutely induce genotoxicity through acute toxicity, bacterial reverse mutation (assessed *via* the Ames test), bone marrow cell micronucleus, and sperm aberration testing in mice. Tzeng et al. (2005) found that following oral administration of water extracts of *Coicis Semen* (1 g/kg body weight), fetal resorptions were significantly increased along with post-implantation mortality, although no embryonic malformation was observed. Notably, spontaneous uterine contractions were significantly increased in pregnant mice. This suggests that water extracts of *Coicis Semen* may cause embryotoxicity; however, this reproductive complication should be investigated further. In addition, in a clinical study conducted by Huang Y. et al. (2016) in which *Coicis Semen* powder was used externally to treat flat warts, individual patients experienced dry skin and scaling, but the symptoms disappeared after discontinuation, suggesting that its clinical application is relatively safe. There is still a lack of clinical observational research on the safety of *Coicis Semen*, and more data are needed in the future, but the above results suggest that *Coicis Semen* has limited toxicity. It is worth noting that there is a plethora of evidence supporting the fact that in the treatment of various malignant tumors, *Coicis Semen* and its extracts can often reduce the toxicity of chemotherapeutic drugs, while improving the therapeutic efficacy.

## 8 Discussion and outlook

*Coicis Semen* is an important botanical drug for the clinical treatment of female reproductive system malignancies, and its efficacy has been confirmed in more than 74 TCM clinical trials. These studies have shown that the administration of prescriptions containing *Coicis Semen* resulted in good therapeutic outcomes in those with cervical cancer, ovarian cancer, endometrial cancer, and other malignant tumors of the female reproductive system by inhibiting the growth of tumors, enhancing immune function, and improving the quality of life. However, based on the existing evidence of *Coicis Semen* pertaining to the treatment of malignant tumors of the female reproductive system, there are still many problems that need to be further adjusted and optimized.

(1) Due to the particularity of clinical research and treatment based on the pattern identification of TCM, most clinical trials included were studies on prescription efficacy with *Coicis Semen* as one of the intervention botanical drugs. Although its efficacy benefits have been confirmed, the main limitations at present include insufficient basis for the compatibility of prescription herbs, lack of theoretical support of TCM in the course of medication, excessively extensive clinical positioning, unclear indication positioning, and deficiencies in the design of aforementioned trials. Consequently, the level of clinical evidence is still weak, and more improved clinical trials are needed in the future.
(2) We found that the component characteristics of *Coicis Semen* extract used in the studies were not completely described, which is lacking in pharmacological research. The reliable pharmacological design, strict implementation, detailed record, and appropriate models and detection methods are required to extensively evaluate the true contribution of *Coicis Semen* and its compounds in the treatment of female reproductive cancers.
(3) The herbal properties of *Coicis Semen* and its compounds have also been studied and analyzed in preclinical experiments. Among the available compounds of *Coicis Semen*, Coix seed oil is currently the most studied active ingredient in the field of cancer research, and preparations have been developed for the clinical treatment of various tumors. Active ingredients, such as coixan polysaccharides and triterpenoids with anti-cancer prospects, are yet to be developed. A growing body of evidence supports the need for further exploration of the role of other active ingredients in *Coicis Semen* in the treatment of female reproductive system malignancies, as well as the synergy between them and existing ingredients.
(4) Currently, only a few reports on the structure modification of active ingredients have been conducted. Nevertheless, future studies to determine novel compounds with good anti-cancer activity through structural modification or synthesis of analogues led by the active ingredients of *Coicis Semen* are warranted.
(5) Due to the lack of relevant studies to determine the specific constituents and action targets, further pharmacokinetic experiments are also required to clarify the absorption, distribution, metabolism, and excretion laws and processes of *Coicis Semen* and its compounds after entering the body.
(6) Although the current body of evidence has shown that toxic side effects caused by *Coicis Semen* are rare, determining a dose-response relationship is essential for the prevention and treatment of diseases, and different doses and drug components may have different anti-cancer effects and protective activities on the female reproductive system. Therefore, further studies are warranted to accurately control the dose of *Coicis Semen* and simultaneously exert the corresponding anti-tumor effects.
(7) With the development of new-generation sequencing technology, high-resolution mass spectrometry technology, multi-omics integrated analyses, and databases, omics technology is developing from traditional single omics to multi-omics. The research of systems biology driven by multi-omics will bring a new paradigm of life science research. In addition to traditional *in vitro* detection, more comprehensive and powerful methods, such as proteomics, genomics, epigenomics, transcriptomics, or metabonomics, will be required to fully understand the overall disturbed biological spectrum of cancer cells affected by the treatment of *Coicis Semen* and its compounds and more systematically and accurately explain the pharmacological mechanism of TCM.
(8) At the present stage, ovarian, cervical, and endometrial cancers are the most reported data in the literature. However, *Coicis Semen* may also be applied to other gynecological malignancies not mentioned in the included literature through the same pathological mechanism based on its TCM theory. This hypothesis needs to be verified by in-depth clinical and experimental research.

In future, the relevant progress based on TCM theory and clinical applications should be combined with phytochemical and pharmacodynamic studies to identify the relevant mechanisms of action and anti-cancer components of *Coicis Semen* and to study the multi-target integration effect for the identification of more lead compounds that could be the foundation for the discovery of new anti-tumor drugs. The studies conducted to date have demonstrated that *Coicis Semen* and its active components exert anti-tumor effects by regulating related signaling pathways; however, it is still necessary to integrate and analyze the mechanism of action through modern research methods to generate credible basic data to inform clinical applications that may lead to better prevention and treatment of female reproductive system malignancies.

## Glossary

ABC: adenosine triphosphate binding cassette
ADP: adenosine diphosphate
AKT: protein kinase B
ATE-EA: adlay testa ethanolic extract
ATE-Hex: hexane fraction of adlay testa ethanolic extracts
AUC: area under the curve
bFGF: basic fibroblast growth factor
CD: cluster of differentiation
CDK4: cyclin-dependent kinase 4
CKLF1: chemokine-like factor 1
CLSE: *Coix lacryma-jobi* var. *ma-yuen* Stapf sprout extract
CNKI: chinese national knowledge infrastructure
COX-2: cyclooxygenase 2
CYP: cytochrome P450
DES/MPA: diethylstilbestrol/medroxyprogesterone 17-acetate
EGFR: epidermal growth factor receptor
ERK: extracellular signal-regulated kinase
HIF-1α-VEGF: hypoxia-inducible factor 1 alpha–vascular endothelial growth factor
HPV: human papillomavirus
IC: icaritin
IC-ME: icaritin and Coix seed oil co-loaded microemulsions
IC-ML: icaritin and Coix seed oil dual-loaded multicomponent thermosensitive lipid complexes
IC-ML(H-): icaritin and Coix seed oil dual-loaded multicomponent thermosensitive lipid complexes incubated at 37°C
IC-ML(H+): icaritin and Coix seed oil dual-loaded multicomponent thermosensitive lipid complexes incubated at 42°C
IκBα: inhibitor of nuclear factor kappa B alpha
IKK: IκB kinase
IL-1β: interleukin 1 beta
IL-6: interleukin 6
KPS: karnofsky performance status
MCP-1: major chemoattractant protein 1
MDR: multidrug resistance
MHC: major histocompatibility complex
MIG-7: migration-induced gene 7
NF-κB: nuclear factor kappa B
NK: natural killer
NMIIA: non-muscle myosin IIA
OS: overall survival
PARP: poly (ADP-ribose) polymerase
PEMT5: protein arginine methyltransferase 5
PFS: progression-free survival
PI3K: phosphoinositide 3-kinase
S100A4: S100 calcium binding protein A4
TCM: traditional Chinese medicine
TNF-α: tumor necrosis factor alpha
UPLC-MS: ultra-high-performance liquid chromatography–mass spectrometry
VEGF: vascular endothelial growth factor
VEGFR-2/KDR: vascular endothelial growth factor receptor-2/kinase-insert domain-containing receptor
