# Supplementary material for: Coicis Semen for the treatment of malignant tumors of the female reproductive system: A review of traditional Chinese medicinal uses, phytochemistry, pharmacokinetics, and pharmacodynamics
Source: Front Pharmacol. 2023 Feb 23;14:1129874. doi: 10.3389/fphar.2023.1129874 (PMC9995914; doi:10.3389/fphar.2023.1129874)
Supplement: Supplementary file 1 [file Table1.DOCX]

Supplementary Material

*Coicis Semen* for the treatment of malignant tumors of the female reproductive system: A review of traditional Chinese medicinal uses, phytochemistry, pharmacokinetics, and pharmacodynamics

Xue Pan, Qian Shen, Chuanlong Zhang, Xiyuan Zhang, Yi Li, Zhuo Chang, and Bo Pang*

*** Correspondence:** Bo Pang: [drpangbo@gmail.com](mailto:drpangbo@gmail.com)

# Supplementary Table S1

**Table S1.** TCM herbs frequently co-prescribed with *Coicis Semen* for the clinical management of malignant tumors of the female reproductive system

| Prescription Name; # of Components | Percentage of *Coicis Semen* in Prescription; Study Design | Type of Disease; Sample Size; Treatment Duration | Treatment Outcomes Assessed and Biomarkers Measured | Main Results (# of Patients) | Ref. |
| --- | --- | --- | --- | --- | --- |
| Fuzheng Jiedu decoction; 19^a^ | 7.27%; Open trial | Cervical cancer; n = 40; 6 months | Response Evaluation Criteria in Solid Tumors (RECIST version 1.1): Complete Response (CR), Partial Response (PR), Stable Disease (SD), or Progressive Disease (PD)  Vascular endothelial growth factor A (VEGF-A) and soluble fms-like tyrosine kinase 1 (sFlt-l) expression; and the numbers of cluster of differentiation 4-positive (CD4^＋^)/cluster of differentiation 8-positive (CD8^＋^) cells and natural killer (NK) cells  Adverse reactions | CR (17) PR (16) SD (5) PD (2) Overall efficacy: 82.5%  Decreased VEGF-A expression, increased sFlt-l expression, and greater numbers of CD4^+^/CD8^+^ and NK cells.  Reduced incidence of adverse reactions | Ma, 2021 |
| Yiqi Huoxue prescription; 16^b^ | 14.35%; Open trial (randomized trial) | Cervical cancer; n = 56; 5 weeks | Response assessment in solid tumors (World Health Organization (WHO) criteria): CR, PR, SD, or PD  Toxicity and side effects  Numbers of CD3^+^, CD4^+^, CD8^+^, and CD4^+^/CD8^+^cells, and the expression of cancer antigen 125 (CA125), carcinoembryonic antigen (CEA), and squamous cell carcinoma antigen (SCCA) | CR (21) PR (23) SD (8) PD (4) Overall efficacy: 78.57%  Reduced incidence of toxicity and side effects involving the digestive, urinary, and circulatory systems  The number of CD3^+^, CD4^+^, and CD4^+^/CD8^+^ cells increased. The expression of CA125, CEA, and SCCA and the number of CD8^+^ cells decreased. | Fan and Zheng, 2021 |
| Fuzheng Jiedu decoction; 19^c^ | 7.27%; Open trial (randomized trial) | Cervical cancer;  n = 49; unknown | Comprehensive therapeutic effect, with responses defined as follows: CR: Disappearance of all lesions and normalization of the levels of tumor-related factors such as VEGF-A and sFlt-1. PR: A decrease of ≥ 50% in the sum of the products of all lesions and a significant decrease in VEGF-A and sFlt-1 expression. NC: A decrease of ≥ 25% or an increase of < 50% in the sum of the products of all lesions, with no significant change in VEGF-A and sFlt-1 expression.  PD: A decrease of < 25% in the sum of the products from all lesions, along with a significant increase in VEGF-A and sFlt-1 expression. | CR (17) PR (22) NC (7) PD (3) Overall efficacy: 81.63% | Zhang, 2020 |
| Huangqi Taohong decoction; 11^d^ | 13.70%; Open trial (randomized trial) | Cervical cancer; n = 58; 12 weeks | Response assessment in solid tumors (WHO criteria): CR, PR, SD, or PD.  Expression of components in the programmed cell death molecule 1 (PD-1)/programmed death molecule-1 ligand (PD-L1) pathway, tumor markers in serum, and T helper cell (Th) cytokines.  Quality of life (QoL) evaluation according to the Functional Assessment of Cancer Therapy-Cervix (FACT-Cx) scale. | CR (1) PR (22) SD (20) PD (15) Disease control rate (DCR): 74.14%  Decreased expression of PD-1 in CD4^+^ T cells, PD-1 in CD8^+^ T cells, and PD-L1 in CD14^+^ monocytes. CEA, CA199, CA125, and squamous cell carcinoma antigen (SCC-Ag) levels decreased, as did the serum expression levels of interferon gamma (IFN-γ), interleukin 2 (IL-2), interleukin 4 (IL-4), and interleukin 6 (IL-6).   Improved QoL inferred from a higher total score using the FACT-Cx scale. | Li et al., 2020 |
| —— 20^e^ | 9.58%;  Open trial (randomized trial) | Cervical cancer; n = 45; 30 days | RECIST version 1.1: CR, PR, SD, or PD  Survival | CR (14) PR (20) SD (8) PD (3) Overall efficacy: 75.55%  The three-year survival rate was 48.44%. | Chen et al., 2020 |
| Fuzheng Yiliu decoction; 14^f^ | 9.30%; Open trial (randomized trial) | Cervical cancer; n = 46; 12 weeks | RECIST version 1.1: CR, PR, SD, or PD  Quantification of T cell subsets  Expression of tumor markers in serum | CR (11) PR (15) SD (8) PD (12) Overall efficacy: 73.91%  Increased numbers of CD3^+^, CD4^+^, and CD4^+^/CD8^+^, and decreased numbers of CD8^+^ cells.  Decreased expression of CEA, osteopontin (OPN), and SCC-Ag. | Zhu and Li, 2019 |
| Fuzheng Quxie prescription; 17^g^ | 10.60%; Open trial (randomized trial) | Cervical cancer; n = 78; 6 weeks | Response assessment in solid tumors (WHO criteria): CR, PR, SD, or PD.  Quantification of T cell subsets.  Functional impairment based on Karnofsky Performance Scale (KPS) scores.   Toxicity and side effects. | CR (31) PR (29) SD (10) PD (8) Overall efficacy: 76.9%  Increased numbers of CD3^+^, CD4^+^, and CD4^+^/CD8^+^ cells, and decreased numbers of CD8^+^ cells.  Increased KPS scores.  Reduced incidence of drug-related toxicity. | Feng et al., 2019 |
| Zhenwu decoction; 12^h^ | 15%; Open trial (randomized trial) | Cervical cancer; n = 67; 48 days | Expression of tumor markers in serum | Decreased expression of CA125, basic fibroblast growth factor (bFGF), and SCC-Ag. | Qiang et al., 2018 |
| Renshen Fuzheng Guben prescription; 12^i^ | 9.71%; Open trial (randomized trial) | Cervical cancer; n = 44; 9 weeks | Response assessment in solid tumors (WHO criteria): CR, PR, SD, or PD.  Functional impairment based on KPS scores.   Toxicity and side effects. | CR (10) PR (13) SD (16) PD (5) Overall efficacy: 52.3%  Increased KPS scores.  Lower incidence of toxicity-related side effects than that of the control group. | Lan, 2018 |
| Fuzheng decoction Ⅰ; 26^j^ | 6.76%; Open trial (randomized trial) | Cervical cancer; n = 32; 6 weeks | Response assessment in solid tumors (WHO criteria): CR, PR, SD, or PD.  Quantification of T cell subsets.  Toxicity and side effects. | CR (10) PR (12) SD (8) PD (2) Overall efficacy: 68.8%  Increased numbers of CD3^+^, CD4^+^, and CD4^+^/CD8^+^ cells, and decreased number of CD8^+^ cells.  Reduced incidence of drug toxicity. | Gong et al., 2018 |
| Xiaozheng Yiai prescription; 14^k^ | 13.33%; Open trial (randomized trial) | Cervical cancer; n = 45; 6 months | Response assessment in solid tumors (WHO criteria): CR, PR, SD, or PD.  Expression of tumor markers in serum. | Overall efficacy: 80%  Decreased expression of SCC-Ag, cytokeratin 19 fragments (CYFRA21-1), B-cell lymphoma 2 (Bcl-2), Survivin, decoy receptor 3 (DcR3), and CEA. | Wang and Yang, 2017 |
| Fuzheng Jiedu decoction; 14^l^ | 13.27%; Open trial (randomized trial) | Cervical cancer; n = 48; 4 weeks | Response assessment in solid tumors (WHO criteria): CR, PR, SD, or PD  QoL based on European Organization for Research and Treatment of Cancer (EORTC) Quality of Life Questionnaire-Core 30 (QLQ-C30) Chinese version 3.0 scores | CR (24) PR (18) SD (6) PD (0) Overall efficacy: 87.5%  Increased EORTC QLQ-Q30 scores. | Liang, 2017 |
| Fuzheng Jiedu decoction; 19^m^ | 7.27%; Open trial | Cervical cancer; n = 34; unknown | Comprehensive therapeutic effect with outcomes defined as follows: CR: Disappearance of all target lesions and a decrease in clinical scores by > 2/3 PR: The product of the two largest diameters of the tumor decreased by ≥50%, clinical score decreased by 1/3–2/3.  SD: Between PR and PD. PD: The product of the two largest diameters of the tumor increases by > 25%, while the clinical score decreases by < 1/3.  Radiation Therapy Oncology Group (RTOG) gastrointestinal toxic effects score.  Expression levels of VEGF-A, sFlt-l, and TNF-α, and the numbers of CD3^+^ and CD4^+^/CD8^+^cells | CR (24) PR (8) SD (2) PD (0) Overall efficacy: 94.12%  Reduced incidence of gastrointestinal toxicity.  VEGF-A expression decreased, whereas sFlt-l and tumor necrosis alpha (TNF-α) expression increased, along with the numbers of CD3^+^, CD4^+^/CD8^+^, and NK cells | Yang et al., 2016 |
| Fuzheng Yiliu decoction; 13^n^ | Unknown; Open trial (randomized trail) | Cervical cancer; n = 23 9 weeks | The expression levels of cluster of differentiation 68 (CD68), cluster of differentiation 83 (CD83), and IL-2 | Increased expression of CD68, CD83, and IL-2. | Huang et al., 2014 |
| Fuzheng Xiaozheng prescription; 14^o^ | 13.33%; Open trial (randomized trial) | Cervical cancer; n = 21; 8 weeks | RECIST version 1.1: CR, PR, SD, or PD  Functional impairment assessed using the KPS, with outcomes defined as follows: Improvement: An increase in KPS scores of ≥ 10 points after treatment compared with pretreatment baseline scores.  Stability: An increase in the KPS score or a decrease of fewer than 10 points after treatment compared with pre-treatment baseline values.  Reduction: A decrease in the KPS score decreased of ≥ 10 points after treatment compared with pretreatment baseline values.  Adverse reactions | CR (0) PR (5) SD (13) PD (3) DCR: 85.7%  Improvement (12) Stability (5) Reduction (4) Overall efficacy of KPS：81%  Reduced incidence of adverse reactions. | Jian et al., 2015 |
| Fuzheng Xiaozheng prescription; 14^p^ | 13.33%; Open trial (randomized trial) | Cervical cancer; n = 22; 8 weeks | Response assessment in solid tumors (WHO criteria): Complete Response (CR), Partial Response (PR), Stable Disease (SD), or Progressive Disease (PD) | CR (8) PR (9) SD (3) PD (2) Overall efficacy: 77.3% | Yin and Huang, 2015 |
| Fuzheng Peiben decoction; 9^q^ | 21.74%; Open trial (randomized trial) | Cervical cancer; n = 45; 3 weeks | Adverse reactions   Quantification of T cell subsets | Reduced incidence of adverse reactions.  Increased numbers of CD4^+^ and CD8^+^ cells, with no significant change in the number of CD4^+^/CD8^+^ cells. | Li and Su, 2015 |
| —— 4^r^ | 36.36%; Open trial | Cervical cancer; n = 36; 9 days | Incidence of pelvic lymphatic cysts (2 months after surgery).  Volume of abdominal drainage.   Incidence of lower limb edema post-surgery | Reduced incidence of pelvic lymphatic cysts.  Decreased abdominal drainage.  No significant difference in the incidence of lower limb edema post-surgery. | Dai et al., 2015 |
| Fuzheng Jiedu decoction; 19^s^ | 7.27%; Open trial (randomized trial) | Cervical cancer; n = 50; unknown | Response assessment in solid tumors (WHO criteria): CR, PR, SD, or PD.  Toxicity based on RTOG gastrointestinal toxicity scores. | CR (35) PR (11) SD (4) PD (0) Overall efficacy: 92%  Reduced incidence of gastrointestinal toxicity. | Chen et al., 2014 |
| —— 6^t^ | 16.13%; Open trial (randomized trial) | Cervical cancer; n = 55; 16 weeks | The numbers of CD3^+^, CD4^+^/CD8^+^, and NK cells, and the expression levels of TNF-α, VEGF-A, and sFlt-1 | The numbers of CD3^+^, CD4^+^/CD8^+^, and NK cells increased, along with the expression of TNF-α. VEGF-A and sFlt-1 expression decreased. | Qin,2013 |
| Jianpi Jiangni decoction; 16^u^ | 8.13%; Open trial (randomized trial) | Cervical cancer; n = 56; 4 weeks | Comprehensive therapeutic effect based on the following criteria: CR: Disappearance of the lesions, clinical symptoms, and adverse reactions; these changes are maintained for > 4 weeks. PR: At least a 30% decrease in the sum of the diameters of target lesions, significant improvement in clinical symptoms and adverse effects after chemotherapy; these changes are maintained for > 4 weeks. SD: Slight reduction in the sum of the diameters of the target lesions and improvement in clinical symptoms and adverse effects after chemotherapy. PD: A significant increase in the sum of the diameters of target lesions, with no improvement of clinical symptoms and adverse effects experienced after chemotherapy. | CR (39) PR (10) SD (5) PD (2) Overall efficacy: 87.5% | Zhao, 2021 |
| Fuzheng Jiedu decoction; 18^v^ | 8%; Open trial (randomized trial) | Cervical cancer; n = 32; unknown | Response assessment in solid tumors (WHO criteria): CR, PR, SD, or PD.  Gastrointestinal toxicity based on RTOG gastrointestinal toxicity scores.  The time until the occurrence of radiation enteritis.  Functional impairment based on KPS scores. | CR (21) PR (9) SD (2) PD (0) Overall efficacy: 93.75%  Reduced incidence of gastrointestinal toxicity.  Prolongation of the time interval until the occurrence of radiation enteritis.  Increased KPS scores. | Guo et al., 2012 |
| —— 5^w^ | Unknown; Open trial (randomized trial) | Cervical cancer; n = 31; 4 weeks | Comprehensive therapeutic effect with outcomes defined as follows: Significant effect: No vaginal secretion or oozing of blood, disappearance of the ulcer surface and/or mass, and the absence of any new lesions. Effective: Significantly reduced vaginal secretion, a reduction of > 50% of the ulcer surface and/or mass, a fading of color, and the absence of new lesions. Ineffective: No significant reduction in vaginal secretions, a reduction in the ulcer surface and/or mass of ≤ 50%, or the appearance of new lesions.  Survival | Significant effect (14) Effective (15) Ineffective (2) Overall efficacy: 93.6%  The one-year survival rate was 67.7%. | Liu et al., 2004 |
| Changpi decoction; 19^x^ | 6.15%; Open trial (randomized trial) | Cervical cancer with radiation proctitis; n = 43; 14 days | Treatment responses were defined as follows:  Significant effect: Disappearance of abdominal pain, diarrhea, blood in the stool, and tenesmus, with a frequency of bowel movements of fewer than twice daily, and normal stool based on routine examination. Effective: Significant resolution of the aforementioned symptoms, with bowel movements occurring 2–4 times daily, and normal stool based on routine examination or weak positivity based on occult blood testing. Ineffective: No significant improvement in the aforementioned symptoms, with a bowel movement frequency fewer than 10 times daily, and no changes in red or white blood cells based on routine stool examinations..  Deterioration: Aggravation of the aforementioned symptoms, with a bowel movement frequency exceeding 10 times daily and an increase in red and white blood cells based on routine stool examination. | Significant effect (26) Effective (14) Ineffective (3) Deterioration (0) Overall efficacy: 93.02% | He et al., 2021 |
| Yichang Cuyu prescription; 9^y^ | 12.12%; Open trial (randomized trial) | Cervical cancer with radiation proctitis; n = 50; 28 days | Comprehensive therapeutic effect assessed based on the following definitions: Significant effect: A rate of reduction of the main traditional Chinese medicine (TCM) syndrome score of > 75%. Bowel movement frequency of ≤ 2 times daily, and normalization of the stool characteristics. Effective: A rate of reduction of the main TCM syndrome score of 50%–75% and a frequency of bowel movements of 3–4 times daily. Ineffective: Failure to satisfy the standard criteria.  Endoscopic curative effect assessed based on the following definitions: Significant effect: Improvement of the intestinal mucosal grading under endoscopy by 2 grades or more after the course of treatment. Effective: Improvement in the intestinal mucosal grading under endoscopy of 1 grade post-treatment. Ineffective: Failure to satisfy the standard criteria above.  QoL based on EORTC-QOL-C30 scores.   Expression of transforming growth factor beta 1 (TGF-β1) in serum.  The incidence of side effects. | Comprehensive therapeutic effect Significant effect (20) Effective (25) Ineffective (5) Overall efficacy: 90%  Endoscopic curative effect Significant effect (25) Effective (17) Ineffective (8) Overall efficacy: 84%  Improved QoL based on increased EORTC-QOL-C30 scores.  Decreased serum TGF-β1 expression.  There was no significant difference in the incidence of side effects between the two groups. | Yang et al., 2019 |
| Changfeng Cuyu decoction; 11^z^ | 10.81%; Open trial (randomized trial) | Cervical cancer with radiation proctitis; n = 54 14 days | Comprehensive therapeutic effect based on the following definitions: Significant effect: Significant relief of gastrointestinal symptoms, with a bowel movement frequency of ≤ 2 times daily and normalization of stool characteristics. Effective: Significant relief of gastrointestinal symptoms, with bowel movements occurring 3–4 times daily. Ineffective: Failure to meet the above standards.  Endoscopic curative effect based on the following definitions: Significant effect: Improvement in the intestinal mucosal grading under endoscopy of 2 grades or more. Effective: Improvement in the intestinal mucosal grading under endoscopy of 1 grade. Ineffective: Failure to meet the above standards.  QoL based on EORTC-QOL-C30 scores.   Expression of IL-1, IL-6, TNF-α, and TGF-β1 in serum.  The incidence of side effects. | Comprehensive therapeutic effect Significant effect (24) Effective (25) Ineffective (5) Overall efficacy: 90.74%  Endoscopic curative effect Significant effect (16) Effective (28) Ineffective (10) Overall efficacy: 81.84%  Improved QoL based on increased EORTC-QOL-C30 scores.  Decreased expression of Serum IL-1, IL-6, TNF-α, and TGF-β.  There was no significant difference in the incidence of side effects between the two groups. | Hu and Huang, 2019 |
| Changfu Kang prescription; 10^aa^ | 12.5%; Open trial (randomized trial) | Cervical cancer with radiation proctitis; n = 50; 14 days | Comprehensive therapeutic effect based on the following definitions: Significant effect: A rate of reduction of the main TCM syndrome score of > 75%, with a bowel movement frequency of ≤ 2 times daily and normalization of stool characteristics. Effective: A rate of reduction of the main TCM syndrome score of 50%–75% and a daily bowel movement frequency of 3–4 times Ineffective: Failure to meet the above standards.  Endoscopic curative effect based on the following definitions: Significant effect: Improvement in intestinal mucosal grading under endoscopy of 2 grades or more after the treatment course. Effective: Improvement in the intestinal mucosal grading under endoscopy of 1 grade after the treatment course. Ineffective: Failure to meet the above standards.  QoL based on EORTC-QOL-C30 scores.   Expression of TGF-β1 in serum.  The incidence of side effects. | Comprehensive therapeutic effect Significant effect (19) Effective (24) Ineffective (7) Overall efficacy: 86%  Endoscopic curative effect Significant effect (23) Effective (22) Ineffective (5) Overall efficacy: 90%  Improved QoL based on increased EORTC-QOL-C30 scores.  Decreased serum TGF-β1 expression.  There was no significant difference in the incidence of side effects between the two groups. | Zhang et al., 2018 |
| —— 16^ab^ | 10.49%; Open trial (randomized trial) | Cervical cancer with radiation proctitis; n = 38; 4 weeks | Comprehensive therapeutic effect based on the following criteria: Significant effect: Resolution of various clinical symptoms, a bowel movement frequency of less than twice daily, firmer stool, and normal mucosa based on colonoscopy results, with no recurrence. after discontinuation of treatment for 2 weeks. Effective: Significant relief of clinical symptoms after treatment, the formation of pseudopolyps or mild inflammation of the mucosa identified via colonoscopy, a bowel movement frequency of 2–4 times daily, and a positive result of stool occult blood testing. Ineffective: No change in clinical symptoms and no improvement based on colonoscopy results. | Significant effect (21) Effective (15) Ineffective (2) Overall efficacy: 94.74% | Huang et al., 2018 |
| Qingre Lishi Jiedu prescription; 10^ac^ | 12.5%; Open trial (randomized trial) | Cervical cancer with radiation proctitis; n = 100; 14 days | Comprehensive therapeutic effect based on the following definitions: Significant effect: A rate of reduction of the main TCM syndrome score of > 75%, with a bowel movement frequency of ≤ 2 times daily and normalization of stool characteristics. Effective: A rate of reduction of the main TCM syndrome score of 50%–75% and a daily bowel movement frequency of 3–4 times. Ineffective: Failure to meet the above standards.  Endoscopic curative effect based on the following definitions: Significant effect: Improvement in intestinal mucosal grading under endoscopy of 2 grades or more after the treatment course. Effective: Improvement in the intestinal mucosal grading under endoscopy of 1 grade after the treatment course. Ineffective: Failure to meet the above standards.  QoL based onEORTC-QOL-C30 scores.   Expression levels of serum TGF-β1.  The incidence of side effects. | Comprehensive therapeutic effect Significant effect (38) Effective (48) Ineffective (14) Overall efficacy: 86%  Endoscopic curative effect Significant effect (46) Effective (44) Ineffective (10) Overall efficacy: 90%  Improved QoL based on increased EORTC-QOL-C30 scores.  Decreased serum TGF-β1 expression.  There was no significant difference in the incidence of side effects between the two groups. | Du et al., 2018 |
| —— 16^ad^ | 10.49%; Open trial (randomized trial) | Cervical cancer with radiation proctitis; n = 42; 4 weeks | Comprehensive therapeutic effect based on the following criteria: Significant effect: Resolution of various clinical symptoms, a bowel movement frequency of less than twice daily, and formed stool with a normal appearance based on routine examinations. Colonoscopy results reveal normal mucosa, and there is no recurrence after discontinuing treatment for 14 days. Effective: Significant relief of clinical symptoms after treatment, with bowel movements occurring 2–4 times daily and a positive result on stool occult blood tests. The formation of pseudopolyps or mild inflammation of the mucosa is detectable via colonoscopy.  Ineffective: No change in clinical symptoms and no evidence of improvement based on colonoscopy results. | Significant effect (27) Effective (14) Ineffective (1) Overall efficacy: 97.62% | Meng and Liu, 2015 |
| —— 11^ae^ | 10.49%; Open trial (randomized trial) | Cervical cancer with radiation proctitis; n = 60; 4 weeks | Efficacy was measured according to the RTOG acute radiation injury grading standards and classified as follows: Cure: Disappearance of intestinal symptoms and a reduction in grading to grade 0. Remission: Significant relief of intestinal symptoms, along with a decrease in grade that fails to reach grade 0. Ineffective: Failure to relieve intestinal symptoms or worsening of symptoms. | Cure (17) Remission (39) Ineffective (4) Overall efficacy: 93.3% | Liu et al., 2015 |
| Changfu Kang prescription; 13^af^ | 9.38%; Open trial (randomized trial) | Cervical cancer with radiation proctitis; n = 32; 2 weeks | Comprehensive therapeutic effect based on the following classifications: Cure: Complete resolution of clinical symptoms and normal stool based on routine examination. Normalization of the intestinal mucosa after colonoscopy, with bowel movements occurring 1–2 times daily. Significant effect: Resolution of various clinical symptoms, no evidence of abnormalities based on routine stool examination, slightly edematous mucosa identified via colonoscopy, and a bowel movement frequency of 2–3 times daily. Effective: Improvement of clinical symptoms and intestinal mucosa lesions based on the results of routine stool examination, with bowel movements occurring 3–4 times daily. Stabilization: Slight improvement or no obvious change in clinical symptoms based on the results of routine examination and colonoscopy. Ineffective: No significant change in clinical symptoms or aggravation or symptoms based on the results of routine stool examination and colonoscopy.  Functional impairment based on the KPS score. | Cure (11) Significant effect (10) Effective (10) Stabilization (1) Ineffective (0) Overall efficacy: 96.9%  Increased KPS scores. | Wang et al., 2014 |
| —— 13^ag^ | 10.53%; Open trial (randomized trial) | Cervical cancer with radiation proctitis; n = 30; 2 weeks | Comprehensive therapeutic effect based on the following criteria: Cure: Resolution of the clinical symptoms after treatment, with a total score decrease of ≥ 95% . Significant effect: Significant improvement in clinical symptoms after treatment, with the total score decreasing by 70%–95%. Effective: Slight improvement of clinical symptoms after treatment, with a decrease in the total score of 30%–70%. Ineffective: No relief from or worsening of clinical symptoms, with the total score decreasing by ≤ 30%. . | Cure (16) Significant effect (5) Effective (7) Ineffective (2) Overall efficacy: 93.3% | Wang et al., 2014 |
| —— 13^ah^ | 14.71%; Open trial (randomized trial) | Cervical cancer with radiation proctitis; n = 60; 1 month | Comprehensive therapeutic effect assessed based on the following criteria: Cure: Resolution of the clinical symptoms and normalization of the damaged mucosa detected via colonoscopy. Significant effect: Resolution of clinical symptoms, evidence of rectal mucosa recovery under colonoscopy, but with congestion and edema being slightly visible in the severely damaged areas. Effective: Relief of clinical symptoms, despite the colonoscopy revealing the continued presence of scattered sheets or spot bleeding. Ineffective: No relief of clinical symptoms after treatment. | Cure (32) Significant effect (16) Effective (8) Ineffective (4) Overall efficacy: 93.3% | Zhuang et al., 2011 |
| —— 10^ai^ | 9.09%; Open trial (randomized trial) | Cervical cancer with radiation proctitis; n = 30; 4 weeks | Comprehensive therapeutic effect based on the following criteria: Cure: Resolution of clinical symptoms and normalization of stool characteristics. Examination via sigmoidoscopy revealing that except for the slightly unclear texture of the intestinal mucosal blood vessels, the rest appear to be normal. Effective: Relief of clinical symptoms and evidence of the normalization of stool characteristics. Sigmoidoscopy reveals mild congestion and edema of the intestinal mucosa, accompanied by a small amount of spot bleeding. Ineffective: No change in clinical symptoms or the results of sigmoidoscopy after treatment compared with pretreatment baseline values. | Cure (20) Effective (7) Ineffective (3) Overall efficacy: 90% | Dong et al., 2011 |
| —— 14^aj^ | 10.17%; Open trial (randomized trial) | Cervical cancer with radiation proctitis; n = 23; 2 weeks | Comprehensive therapeutic effect based on the following criteria: Cure: Disappearance of clinical symptoms after treatment, with the total score decreasing by ≥ 95% . Significant effect: Significant improvement of clinical symptoms after treatment, with the total score decreasing by 70%–95%. Effective: Slight improvement of clinical symptoms after treatment, with the total score decreasing by 30%–70%. Ineffective: No relief or worsening of clinical symptoms, with the total score decreasing by ≤ 30%. | Cure (12) Significant effect (4) Effective (5) Ineffective (2) Overall efficacy: 91.3% | Zhang, 2010 |
| —— 16^ak^ | 10.49%; Open trial (randomized trial) | Cervical cancer with radiation proctitis; n = 21; 2 weeks | Comprehensive therapeutic effect based on the following criteria:  Cure: Disappearance of clinical symptoms after treatment, with the total score decreasing by ≥ 95% and with the endoscopy results appearing normal. Significant effect: Significant improvement of clinical symptoms after treatment, with the total score decreasing by 70%–95%. Effective: Slight improvement of clinical symptoms after treatment, with the total score decreasing by 30%–70%, along with improved endoscopy results. Ineffective: No relief or worsening of clinical symptoms or endoscopy results, with the total score decreasing by ≤ 30%. | Cure (13) Significant effect (4) Effective (3) Ineffective (1) Overall efficacy: 95.2% | Zhang and Cui, 2010 |
| —— 10^al^ | 10.42%; Open trial (randomized trial) | Prevention of cervical cancer radiation proctitis; n = 27; Until the end of radiotherapy | The incidence of radiation proctitis. | The incidence of radiation proctitis was 7.4% (vs 59.3% in the control group). | Chen, 2019 |
| Qinghua Guchang decoction; 17^am^ | 8.30%; Open trial (randomized trial) | Prevention of cervical cancer radiation proctitis; n = 45; 30 days | RTOG/EORTC grading scale.  QoL based on the KPS score.  Outcomes were classified according to the following criteria: Improvement: An increase in the KPS score of ≥ 10 points after treatment compared with the pre-treatment baseline values.  Stability: An increase or decrease in the KPS score of fewer than 10 points after treatment compared with pre-treatment baseline values.  Reduction: A reduction in the KPS score of ≥ 10 points after treatment compared with pre-treatment baseline values. | Defecation grading three months after treatment: Grade 0（22）  Grade 1（11） Grade 2（8） Grade 3（4） Grade 4（0）  The improvement rate and stability rate (efficacy of KPS score) accounted for 88.89% (vs 74.42% in the control group). | Wang and Ye, 2009 |
| —— 10^an^ | 8.30%; Open trial (randomized trial) | Prevention of cervical cancer radiation proctitis; n = 43; Until the end of radiotherapy | The incidence of radiation proctitis. | The incidence of radiation proctitis was 7.5% (vs 28.2% in the control group). | Dong et al., 2012 |
| Tiaogan Jianpi Jiedu decoction; 16^ao^ | 8.13%; Open trial (randomized trial) | Prevention of cervical cancer radiation proctitis; n = 64; unknown | The incidence of radiation proctitis.   The frequency and dose of exposure in those experiencing a rectal radiation reaction.  The incidence of diarrhea and abdominal pain. | The incidence of radiation proctitis was 32.81% (vs 53.12% in the control group).  The frequency and dose of exposure were higher than those in the control group.  The incidence of diarrhea and abdominal pain were 31.3% and 32.8%, respectively (vs 50% and 53.1%, respectively in the controls). | Ma et al., 2011 |
| Shenling Baizhu powder; 13^ap^ | 16.23%; Open trial (randomized trial) | Prevention of gynecological malignancy (cervical cancer, endometrial cancer) radiation proctitis; n = 30 35–38 days | Associated symptoms (stool frequency (3–4 times daily), mucous in stool, and hypogastralgia).  The incidence of radiation-induced rectal injury. | Decreased incidence of associated symptoms.  The incidence of radiation rectal injury was 43.3% (vs 96.2% in the control group). | Hu et al., 2005 |
| —— 14^aq^ | 8.52%; Open trial (randomized trial) | Pelvic lymphatic cyst in patients with cervical cancer; n = 45; 2 weeks | Comprehensive therapeutic effect based on the following criteria: Cure: Complete resolution of symptoms after treatment, with’ pelvic ultrasound revealing no abnormalities. Effective: Disappearance of’ clinical symptoms after treatment, with pelvic ultrasound revealing a significant reduction of the size of the cyst. Ineffective: No improvement or worsening of symptoms after treatment, with pelvic ultrasound revealing no change in lymphatic cysts and no reduction in pressure-induced pain. | Cure (31) Effective (12) Ineffective (2) Overall efficacy: 95.56% | Qi and Duan, 2014 |
| Xiaozheng Lishi prescription; 16^ar^ | 6.35%; Open trial | Pelvic lymphatic cyst in patients with cervical cancer; n = 28; 2 weeks | Comprehensive therapeutic effect based on the following criteria: Cure: Disappearance of clinical symptoms and signs after one course of treatment (7 days). Significant effect: Disappearance of clinical symptoms and a significant reduction in cysts after two courses of treatment (14 days). Ineffective: No significant reduction in clinical symptoms, no significant reduction in the size of the lymphatic cyst, and a slight or no reduction in pressure-induced pain. | Cure (15) Significant effect (11) Ineffective (2) Overall efficacy: 92.86% | Zhao, 2013 |
| —— 14^as^ | 9.52%; Open trial (randomized trial) | Pelvic lymphatic cyst in patients with cervical cancer; n = 37; 20 days | Comprehensive therapeutic effect based on the following definitions: CR: Disappearance of pain, a lessening of lower abdominal discomfort, and the disappearance of cysts. PR: Significant pain relief, a lessening of lower abdominal discomfort, and a reduction in cysts. No response (NR): No significant improvement in symptoms and signs. | CR (21) PR (14) NR (2) Overall efficacy: 94.6% | Han et al., 2013 |
| —— 14^at^ | 7.94%; Open trial (randomized trial) | Pelvic lymphatic cyst in patients with cervical cancer; n = 28; 14 days | Comprehensive therapeutic effect based on the following criteria: Cure: Disappearance of clinical symptoms and signs after treatment. Significant effect: Disappearance of clinical symptoms and a significant reduction in cysts after treatment. Ineffective: No significant reduction in clinical symptoms, no significant reduction in the size of the lymphatic cyst, and a slight or no reduction in pressure-induced pain. | Cure (20) Significant effect (7) Ineffective (1) Overall efficacy: 96.4% | Fu and Lei, 2000 |
| —— 17^au^ | 13.95%; Open trial | Urinary retention in cervical cancer; n = 30; unknown | The recovery time of voluntary voiding function and residual urine volume of the bladder.  The incidence of urinary tract infections. | The recovery time following voluntary voiding and the residual urine volume of the bladder were significantly lower in the treatment group than in the control group.  The incidence of urinary tract infection was 6.78% (vs 23.33% in the control group). | Yang et al., 2017 |
| Buzhong Yiqi decoction; 9^av^ | 20.69%; Open trial (randomized trial) | Urinary retention in cervical cancer; n = 45; 2 weeks | Comprehensive therapeutic effect based on the following criteria: Cure: After treatment, the patient can urinate freely without obstruction, and the residual urine volume is < 100 mL. The patient can urinate automatically, and the clinical symptoms disappear. Effective: Patients occasionally experience dribbling urination, with a residual urine volume of < 100 mL. The patient can urinate spontaneously, and clinical symptoms have improved. Ineffective: Patients with a residual urine volume of > 100mL and are still unable to urinate automatically, and the clinical symptoms do not improve or even worsen.  The duration of indwelling urinary catheter use and residual urine volume. | Cure (21) Effective (15) Ineffective (9) Overall efficacy: 80%  Decreased duration of indwelling urinary catheter use and reduced residual urine volume. | Liu, 2016 |
| Bushen Yiqi Tongli prescription; 18^aw^ | 13.33%; Self-control study | Urinary retention in cervical cancer; n = 9; 1 week | The residual bladder urine volume.   The recovery time of voluntary voiding function and recurrence. | Decreased residual bladder urine volume.  The recovery time of voluntary voiding function was (16.22±6.12) days. After 3–6 months of follow-up, all patients were free of recurrence. | Liang et al., 2015 |
| Huazhi decoction; 10^ax^ | 27.78%; Open trial (randomized trial) | Diarrhea in cervical cancer; n = 30 1 week | Comprehensive therapeutic effect based on the following criteria: Cure: Soft or formed stools, occurring less than 3 times a day. Effective: Unformed stools, but voiding is performed significantly less frequently than before treatment at 3–5 times a day. Ineffective: No improvement in symptoms, even combined with infection or water-electrolyte imbalance. | Cure (14) Effective (9) Ineffective (7) Overall efficacy: 76.67% | Song et al., 1998 |
| Xiaoliu Yiai prescription; 10^ay^ | 8.62%; Open trial (randomized trial) | Gastrointestinal protection in patients with cervical cancer; n = 45; 6 months | Levels of oxidative stress markers in serum.   Quantification of T cell subsets.  QoL based on the EuroQol Five Dimensions Questionnaire (EQ-5D).  Incidence of gastrointestinal symptom (nausea, vomiting, and diarrhea). | Increased expression of superoxide dismutase (SOD) and the number of CD4^+^, and CD4^+^/CD8^+^ cells.  Decreased expression of malondialdehyde (MDA) and the number of CD8^+^ cells.  Increased scores of the Mobility, Pain/Discomfort, and Anxiety/Depression subscales of the EQ-5D.  Decreased nausea, vomiting, and diarrhea. | Yang et al., 2017 |
| Wuling powder and Wupi drink; 14^az^ | 6.03%; Open trial (randomized trial) | Lymphedema after gynecological tumor surgery; n = 43; 6 weeks | Comprehensive therapeutic effect based on the following criteria: Significant effect: Complete disappearance of symptoms and signs of lymphedema in the lower extremities, with limb functions returning to normal. Effective: Partial disappearance of symptoms and signs of lower limb lymphedema and partial recovery of limb function. Ineffective: No change or a slight increase in the original edema site.  Circumference of the affected limb. | Significant effect (26) Effective (12) Ineffective (5) Overall efficacy: 88.37%  Reduced circumference of the affected limb at mid-calf, upper ankle, and mid-thigh. | Wang et al., 2021 |
| —— 11^bb^ | 12.12%; Open trial (randomized trial) | Ovarian cancer; n = 46; 6 months | RECIST version 1.1: PR, SD, or PD.  Quantification of T cell subsets. | CR (5) PR (23) SD (13) PD (5) Overall efficacy: 60.87%  Increased numbers of CD3^+^, CD4^+^, and CD4^+^/CD8^+^ cells, but a decreased number of CD8^+^ cells. | Zhang et al., 2018 |
| Shenling Baizhu powder; 15^bc^ | 12.5%; Open trial (randomized trial) | Ovarian cancer; n = 46; 7 weeks | Gastrointestinal function.  Clinical symptom scores (abdominal pain, bloating, gastrointestinal response scores).  Serum inflammatory factor levels (INF-α, IL-6, and C-reactive protein (CRP)). | Shortened recovery time of bowel sounds, first anal exhaust time, and first anal defecation time.  Decreased abdominal pain, bloating, and gastrointestinal response scores.  Decreased expression of INF-α, IL-6, and CRP. | Wang et al., 2018 |
| Yiqi Jianpi Yangxue decoction; 11^bd^ | 17.96%; Open trial (randomized trial) | Ovarian cancer; n = 28; 8 weeks | Comprehensive therapeutic effect based on the following criteria: Superior: Disappearance of all measurable lesions after treatment and maintenance of CA125 concentrations of ≤ 35 U/mL for more than 4 weeks. Good: Measurable degree of lesion disappearance and a reduction in CA125 expression of ≥ 50% after treatment, with the effects maintained for more than 4 weeks. Poor: Less than 50% reduction in lesion size and CA125 expression.  Incidence of adverse reactions.  Generic Quality of Life Inventory-74 (GQOL-74) score. | Comprehensive therapeutic effect Superior (11) Good (16) Poor (1) Overall efficacy: 96.43%  Occurrence of adverse reactions Hair loss (1) Numbness of extremities (1) Impaired liver function (0) Weakness of extremities (1) The incidence of adverse reactions was 10.71%.  Increased GQOL-74 scores . | Pan, 2018 |
| —— 14^be^ | 11.76%; Open trial (randomized trial) | Ovarian cancer; n = 34; 24 weeks | Survival | The 1-year survival rate was 100% (vs 94.1%) The 2-year survival rate was 88.2% (vs 85.3%) The 3-year survival rate was 79.4% (vs 64.7%) The 4-year survival rate was 61.8% (vs 41.2%) The 5-year survival rate was 17.6% (vs 2.9%) | Li, 2018 |
| Yiqi Jianpi Yangxue decoction; 13^bf^ | 14.93%; Open trial (randomized trial) | Ovarian cancer; n = 21; 12 weeks | Response assessment in solid tumors (WHO criteria): CR, PR,SD, or PD.  Functional impairment based on KPS scores.  Toxicity and side effects. | CR (2) PR (14) SD (4) PD (1) Overall efficacy: 76.19%  Increased KPS scores.  Reduced incidence of gastrointestinal adverse reactions. | Xuan and Ge, 2011 |
| Shenqi Fuzheng Baidu pill; 14^bg^ | 6.67%; Open trial (randomized trial) | Ovarian cancer; n = 120; 4–6 weeks | The chemotherapy completion rate.   Changes in lesion characteristics.  Survival. | The chemotherapy completion rate was 88.33% (vs 67.5%).  The disease remission rate (CR+PR) was 54.17% (vs 30.00%).  The 1-year survival rate was 81.45% (vs 78.58%). The 3-year survival rate was 65.46% (vs 45.38%). The 5-year survival rate was 30.65% (vs 28.73%). | Yu et al., 2000 |
| —— 14^bh^ | 10.34%; Open trial | Ovarian cancer; n = 27; 4 weeks | Response assessment in solid tumors (WHO criteria): CR, PR, SD, PD.  Completion rate of chemotherapy and the incidence of toxic side effects.  Survival. | CR (15) PR (8) SD (2) PD (2) Overall efficacy: 85.19%  The chemotherapy completion rate was 81.48% (22/27). Mild gastrointestinal reactions were observed in 18 patients, although no other toxic side effects were observed.  The 1-year survival rate was 81.84% . The 5-year survival rate was 50.09%. | Xia and Liu, 1999 |
| Zhenwu decoction; 12^bi^ | 14.08%; Open trial (randomized trial) | Ovarian cancer; n = 43 6 weeks | Comprehensive therapeutic effect based on the following criteria: Cure: After surgery, the lesion area is basically healed, allowing the patient to walk and eat normally. Improvement: Slight pain still present after surgery, but it does not affect normal movement. Significant effect: No infection at the surgical incision, but more painful. Ineffective: No significant change before and after treatment.  QoL based on KPS scores. Improvement: KPS score increased by ≥ 10 points after treatment compared with pre-treatment baseline values.  Stability: KPS score increased or decreased by less than 10 points after treatment compared with pre-treatment baseline values.  Reduction: KPS score decreased by ≥ 10 points after treatment compared with pre-treatment baseline values. | Comprehensive therapeutic effect Cure (16) Improvement (12) Significant effect (10) Ineffective (5) Overall efficacy: 88.37%  The improvement rate and stability rate accounted for 72.09% (vs 27.91% in the control group). | Wang and Gao, 2015 |
| Fuzheng Xiaoliu decoction; 14^bj^ | 9.84%; Open trial (randomized trial) | Ovarian cancer; n = 43; 30 days | Comprehensive therapeutic effect based on the following criteria: CR: Disappearance of ovarian malignant tumors and no new tumor lesions formed within 30 days. PR:A decrease in the tumor volume of ≥ 50% after treatment and no new tumor lesions formed within 30 days. SD: A decrease in tumor volume of < 50% or an increase of < 25% after treatment. PD: Increase in tumor volume of > 25% after treatment, with new tumor lesions forming within 30 days. | CR (9) PR (13) SD (14) PD (7) Overall efficacy: 51.2% | Chen et al., 2014 |
| Shenling Baizhu flavoring powder; 15^bk^ | 12.5%; Open trial (randomized trial) | Ovarian cancer; n = 45; 1 week | Levels of inflammatory factors and sex hormones in serum. | Decreased levels of serum CRP, IL-6, TNF-α, follicle-stimulating estrogen (FSH), estradiol (E2), and luteinizing hormone (LH). | Huang et al., 2020 |
| Shuyu pill; 21^bl^ | 6.70%; Open trial (randomized trial) | Ovarian cancer; n = 30; 90 days | Functional impairment based on KPS scores   Expression levels of serum tumor markers. | Increased KPS scores.  Decreased expression of CA125, CA199, CEA, and human epididymis protein 4 (HE4). | Tang, 2019 |
| Yishen Huayu Xiaozheng prescription; 17^bm^ | 12%; Open trial (randomized trial) | Ovarian cancer; n = 30; 21 days | RECIST version 1.1: CR, PR, SD, and PD  Expression levels of tumor markers in serum. | CR (0) PR (5) SD (18) PD (7) (DCR) :76.67%  Objective response rate (ORR): 16.67%  Decreased CA125 and CEA expression levels. | Wang, 2020 |
| Fuzheng Xiaoliu decoction; 14^bn^ | 10.34%; Open trial (randomized trial) | Ovarian cancer; n = 43; 3–4 weeks | Comprehensive therapeutic effect based on the following criteria: CR: Disappearance of ovarian malignant tumors and no formation of new tumor lesions within 30 days. PR: Decrease in tumor volume of ≥ 50% after treatment, with no new tumor lesions forming within 30 days. SD: Decrease in tumor volume of < 50% or an increase of < 25% after treatment. PD: Increase in tumor volume of > 25% after treatment, with new tumor lesions forming within 30 days. | CR (11) PR (19) SD (9) PD (4) Overall efficacy: 69.8% | Jin and Kong, 2015 |
| Shenqi Fuzheng Baidu pill; 14^bo^ | 6.67%; Open trial (randomized trial) | Ovarian cancer; n = 66; 2 months | Comprehensive therapeutic effect based on the following criteria: Significant effect: Disappearance of clinical symptoms and signs, disappearance of the tumor for more than one month, and a decrease in the maximum diameter of the lesions and its products by ≥ 50%. Effective: Improvement in clinical symptoms and signs, with a decrease in the maximum diameter of the lesions and its products by < 50%, along with an .increase of < 25% within one month. Ineffective: No change in clinical symptoms or signs or even deterioration, less than 25% reduction of lesions or the discovery of new lesions. | Significant effect (40) Effective (20) Ineffective (6) Overall efficacy: 90.91% | Li et al., 2014 |
| —— 16^bp^ | 4.44%; Open trial (randomized trial) | Ovarian cancer; n = 48; 32 weeks | Response assessment in solid tumors (WHO criteria): CR, PR, SD, and PD | CR (30) PR (12) SD+ PD (9) Overall efficacy: 87.5% | Li et al., 2006 |
| Wenyang Lishui prescription 16^bq^ | 7.84%; Open trial (randomized trial) | Ovarian cancer ascites; n = 44; 8 weeks | Comprehensive therapeutic effect based on the following criteria: CR: The absence of a liquid dark area on ultrasound, complete disappearance of clinical symptoms, and the presence of fluid in the abdominal cavity for a period exceeding 4 weeks. PR: Decreased depth of the liquid dark area detected via ultrasonography of > 50%, a decrease in the amount of fluid accumulation by > 50%, and improvement of clinical symptoms. SD: A decrease of < 50% in the depth of the liquid dark area detected through ultrasonography, accompanied by a decrease in the amount of fluid accumulation by < 50%, and the partial resolution of clinical symptoms. PD: Failure to control the fluid accumulation or worsening of the condition, with the volume of fluid accumulation increasing significantly. The clinical symptoms do not improve, and drainage and decompression are still required within 4 weeks.  Quantification of T cell subsets.  Functional impairment based on KPS scores. | CR (20) PR (15) SD (5) PD (4) DCR: 91.0%  Response rate (RR): 79.5%  Increased numbers of CD3^+^, CD4^+^, and CD4^+^/CD8^+^ cells.  Increased KPS scores. | Zhang et al., 2018 |
| Yishen Kangliu prescription; 14^bt^ | 9.09%; Open trial (randomized trial) | Ovarian cancer ascites; n = 49; 8 weeks | Efficacy of ascites control Significant effect: Complete disappearance of ascites; an effect that is maintained for more than 30 days. Effective: A decrease in the ascites by > 50% after treatment, an effect that is maintained for 30 days without increasing. Ineffective: A decrease in ascites by < 50% after treatment or even an increase.  Functional impairment based on KPS scores.   Expression levels of tumor markers.  Quantification of T cell subsets and NK cells. | Significant effect (20) Effective (22) Ineffective (7) Overall efficacy: 85.7%  Increased KPS scores.  Decreased expression of CEA, CA125, HE4, and VEGF.  Increased numbers of CD3^+^, CD4^+^, CD4^+^/CD8^+^, and NK cells. | Chen et al., 2018 |
| Shenling Baizhu flavoring powder; 12^bs^ | 17.65%; Open trial (randomized trial) | Ovarian cancer ascites; n = 30; 4 weeks | Efficacy of ascites control based on the following criteria: CR: Complete disappearance of ascites, a change that is maintained for more than 4 weeks PR: A decrease in the ascites by > 50% after treatment, an effect that is maintained for 4 weeks without increasing. NC: A decrease in the ascites by < 50% or an increase of < 25%. PD: Significant increase in peritoneal effusion. | CR (8) PR (18) NC (3) PD (1) Overall efficacy: 86.7% | Yi et al., 2017 |
| —— 11^bt^ | 13.30%; Open trial | Ovarian cancer ascites; n = 42; unknown | Comprehensive therapeutic effect based on the following criteria: CR: Disappearance of both clinical symptoms and ascites for more than 4 weeks. PR: A decrease in the depth of the liquid dark area detected via ultrasonography by > 50% , along with a significant reduction in ascites for 4 weeks. SD: A decrease of < 50% in the depth of the liquid dark revealed by ultrasonography, along with some reduction in ascites. PD: A significant increase in abdominal fluid volume.  Functional impairment based on KPS scores. | CR (32) PR (5) SD (2) PD (3) Overall efficacy: 88.10%  Increased KPS) score increased. | Sun, 2016 |
| —— 17^bu^ | 4.65%; Open trial (randomized trial) | Ovarian cancer ascites; n = 33; unknown | Comprehensive therapeutic effect based on the following criteria: CR: Disappearance of symptoms and the mass detected during CT examination, with no effusion detected on ultrasound examination, and lasting more than 8 weeks after the end of treatment. PR: Significant improvement in symptoms after treatment, along with a significant reduction in the size of the mass detected on CT and a > 50% decrease in effusion lasting at least 8 weeks. SD: No significant change in the size of the mass detected via CT, but a < 50% decrease in effusion, maintained for at least 8 weeks PD: Increased symptoms along with > 25% enlargement of the ovarian mass.  Survival | CR (6) PR (21) SD (5) PD (1) Overall efficacy: 81.82%  The 5-year survival rate was 54.55% (vs 23.81% in the control group). | Yuan and Chen, 2012 |
| —— 11^bv^ | 13.30%; Open trial (randomized trial) | Ovarian cancer ascites; n = 30; 15 days | CR: Complete disappearance of ascites and maintenance of the effect for more than 1 month. PR: A decrease in ascites volume by > 50% and maintenance of the change for more than 1 month. Ineffective: A decrease in the ascites volume by < 50% or a short-term increase in volume.  Functional impairment based on KPS scores . | CR (7) PR (19) Ineffective (4) Overall efficacy: 86.7%  Increased KPS scores. | Jin et al., 2011 |
| Flavoring Shenling Baizhu flavoring powder; 15^bw^ | 12.5%; Open trial (randomized trial) | Ovarian cancer ascites; n = 20; 3 weeks | Efficacy of ascites control.  Functional impairment based on KPS scores. | Decreased the depth of ascites.  Increased KPS scores. | Hao, 2011 |

Abbreviations: Bcl-2, B-cell lymphoma 2; bFGF, basic fibroblast growth factor; CA125, cancer antigen 125; CD, cluster of differentiation; CEA, carcinoembryonic antigen; CR, complete response; CRP, C-reactive protein; CYFRA21-1, cytokeratin 19 fragments; DCR, disease control rate; DcR3, decoy receptor 3; E2, estradiol; EQ-5D, EuroQol Five Dimensions Questionnaire; EORTC, European Organization for Research and Treatment of Cancer; FACT-Cx, Functional Assessment of Cancer Therapy-Cervix Cancer; FSH, follicle-stimulating hormone; GQOL-74, Generic Quality of Life Inventory-74; HE4, human epididymis protein 4; IFN-γ, interferon gamma; IL-1β, interleukin 1 beta; KPS, Karnofsky Performance Scale; LH, luteinizing hormone; MDA, malondialdehyde; NC, no change; NK, natural killer; OPN, osteopontin; PD, progressive disease; ORR, objective response rate; PD-1, programmed cell death molecule; PD-L1, programmed death molecule-1 ligand; PR, partial response; QLQ-C30, Quality of Life Questionnaire-Core 30; QoL, quality of life; RECIST, Response Evaluation Criteria in Solid Tumors; RR, response rate; RTOG, Radiation Therapy Oncology Group; SCCA, squamous cell carcinoma antigen SD, stable disease; sflt-1, soluble fms-like tyrosine kinase 1; SOD, superoxide dismutase; TCM, traditional Chinese medicine; Th, T helper; TNF-α, tumor necrosis factor alpha; VEGF-A, vascular endothelial growth factor A; WHO, World Health Organization.

^a^ *Hedyotis Diffusae Herba* 15g, *Sophorae Flos* 15g, *Scutellariae Barbatae Herba* 15g, *Psoraleae Fructus* 10g, *Phellodendri Chinensis Cortex* 10g, *Rehmanniae Radix* 15g, *Myristicae Semen* 10g, *Lablab Semen Album* 15g, ***Coicis Semen* 20g**, *Cimicifugae Rhizoma* 10g, *Puerariae Lobatae Radix* 10g, *Poria* 15g, *Paeoniae Radix Rubra* 10g, *Paeoniae Radix Alba* 15g, *Dioscoreae Rhizoma* 15g, *Ganoderma* 15g, *Astragali Radix* 30g, *Salviae Miltiorrhizae Radix et Rhizoma* 15g, *Pseudostellariae Radix* 15g.

^b^ *Astragali Radix* 30g, *Codonopsis Radix* 30g, ***Coicis Semen* 30g,** *Angelicae Sinensis Radix* 15g, *Lycii Fructus* 15g, *Curcumae Rhizoma* 15g, *Poria* 15g, *Hedyotis Diffusae Herba* 15g, *Cinnamomi Ramulus* 15g, *Atractylodis Macrocephalae Rhizoma* 10g, *Fritillariae Cirrhosae Bulbus* 10g, *Prunellae Spica* 10g, *Arnebiae Radix* 10g, *Glycyrrhizae Radix et Rhizoma* 8g, *Sparganii Rhizoma* 6g, *Buthus martensi* 5g.

^c^  *Psoraleae Fructus* 10g, *Puerariae Lobatae Radix* 10g, *Cimicifugae Rhizoma* 10g, *Phellodendri Chinensis Cortex* 10g, *Myristicae Semen* 10g, *Paeoniae Radix Rubra* 10g, *Hedyotis Diffusae Herba* 15g, *Scutellariae Barbatae Herba* 15g, *Poria* 15g, *Rehmanniae Radix* 15g, *Sophorae Flos* 15g, *Lablab Semen Album* 15g, *Pseudostellariae Radix* 15g, *Salviae Miltiorrhizae Radix et Rhizoma* 15g, *Ganoderma* 15g, *Dioscoreae Rhizoma* 15g, *Paeoniae Radix Alba* 15g, ***Coicis Semen* 20g,** *Astragali Radix* 30g.

^d^  *Poria* 15g, *Pseudostellariae Radix* 15g, *Astragali Radix* 30g, ***Coicis Semen* 20g,** *Paeoniae Radix Rubra* 12g, *Carthami Flos* 13g, *Persicae Semen* 10g, *Angelicae Sinensis Radix* 10g, *Atractylodis Macrocephalae Rhizoma* 10g, *Citri Reticulatae Pericarpium* 6g, *Glycyrrhizae Radix et Rhizoma* 6g.

^e^  *Astragali Radix* 30g, ***Coicis Semen* 30g,** *Polygonati Rhizoma* 15g, *Spatholobi Caulis* 15g, *Jujubae Fructus* 15g, *Rehmanniae Radix Praeparata* 15g, *Dioscoreae Rhizoma* 15g, *Paeoniae Radix Alba* 15g, *Poria* 15g, *Ziziphi Spinosae Semen* 15g, *Longan Arillus* 15g, *Adenophorae Radix* 15g, *Lycii Fructus* 12g, *Angelicae Sinensis Radix* 12g, *Cuscutae Semen* 12g, *Agrimoniae Herba* 12g, *Atractylodis Macrocephalae Rhizoma* 12g, *Asini Corii Colla* 9g, *Pinelliae Rhizoma* 9g, *Chuanxiong Rhizoma* 10g.

^f^  *Astragali Radix* 15g, *Scutellariae Barbatae Herba* 20g, *Hedyotis Diffusae Herba* 30g, *Curcumae Rhizoma* 10g, *Codonopsis Radix* 20g, *Atractylodis Macrocephalae Rhizoma* 10g, *Poria* 15g, *Aurantii Fructus* 10g, *Magnoliae Officinalis Cortex* 10g, *Mume Flos* 10g, *Polygoni Cuspidati Rhizoma et Radix* 15g, ***Coicis Semen* 20g,** *Amomi Fructus* 10g, *Setariae Fructus Germinatus* 20g.

^g^  *Astragali Radix* 30g, *Codonopsis Radix* 30g, *Hedyotis Diffusae Herba* 30g, ***Coicis Semen* 30g,** *Angelicae Sinensis Radix* 15g, *Poria* 15g, *Curcumae Longae Rhizoma* 15g, *Rhei Radix et Rhizoma* 15g, *Ligustri Lucidi Fructus* 15g, *Lycii Fructus* 15g, *Curcumae Rhizoma* 15g, *Cuscutae Semen* 10g, *Prunellae Spica* 10g, *Atractylodis Macrocephalae Rhizoma* 10g, *Fritillariae Cirrhosae Bulbus* 10g, *Arnebiae Radix* 10g, *Glycyrrhizae Radix et Rhizoma* 8g.

^h^  *Aconiti Lateralis Radix Praeparata* 10g, *Aurantii Fructus* 10g, *Persicae Semen* 10g, *Zingiberis Rhizoma Recens* 15g, *Astragali Radix* 15g, *Atractylodis Macrocephalae Rhizoma* 15g, P*aeoniae Radix Alba* 15g, *Pinelliae Rhizoma* 15g, *Citri Reticulatae Pericarpium* 15g, *Poria* 20g, *Leonuri Herba* 30g, ***Coicis Semen* 30g.**

^I^  *Ginseng Radix et Rhizoma* 25g, *Ophiopogonis Radix* 10g, *Rehmanniae Radix Praeparata* 15g, *Angelicae Sinensis Radix* 20g, 6 of *Jujubae Fructus*, *Fritillariae Cirrhosae Bulbus* 15g, *Astragali Radix* 30g, *Paeoniae Radix Alba* 20g, *Curcumae Rhizoma* 15g, *Citri Reticulatae Pericarpium* 15g, ***Coicis Semen* 20g,** *Glycyrrhizae Radix et Rhizoma* 6g.

^j^  *Rehmanniae Radix Praeparata* 20g, *Corni Fructus* 30g, *Dioscoreae Rhizoma* 15g, *Poria* 20g, *Moutan Cortex* 9g, *Alismatis Rhizoma* 15g, *Taxilli Herba* 15g, *Lycii Fructus* 10g, ***Coicis Semen* 30g,** *Atractylodis Macrocephalae Rhizoma* 10g, *Pinelliae Rhizoma* 9g, *Rehmanniae Radix* 10g, *Glehniae Radix* 20g, *Salviae Miltiorrhizae Radix et Rhizoma* 10g, *Angelicae Sinensis Radix* 10g, *Curcumae Rhizoma* 12g, *Paeoniae Radix Alba* 15g, *Scutellariae Barbatae Herba* 20g, *Pseudobulbus Cremastrae seu Pleiones* 20g, *Duchesnea indica Focke* 20g, *Solanum Nigrum* 20g, *Crataegi Fructus* 30g, *Hordei Fructus Germinatus* 30g, *Massa Dermentata Medicinalis* 30g, *Citri Reticulatae Pericarpium* 8g, *Glycyrrhizae Radix et Rhizoma* 6g.

^k^  *Astragali Radix* 30g, ***Coicis Semen* 30g,** *Scutellariae Barbatae Herba* 30g, *Ligustri Lucidi Fructus* 15g, *Codonopsis Radix* 15g, *Atractylodis Macrocephalae Rhizoma* 15g, *Smilacis Glabrae Rhizoma* 15g, *Curcumae Rhizoma* 15g, *Cyathulae Radix* 15g, *Cuscutae Semen* 10g, *Paridis Rhizoma* 10g, *Lycii Fructus* 10g, *Gekko Japonicus Dumeril et Bibron* 10g, *Glycyrrhizae Radix et Rhizoma* 5g.

^l^  *Astragali Radix* 30g, *Codonopsis Radix* 20g, *Rhei Radix et Rhizoma* 10g, *Curcumae Longae Rhizoma* 15g, *Fritillariae Thunbergii Bulbus* 10g, *Atractylodis Macrocephalae Rhizoma* 10g, ***Coicis Semen* 30g,** *Lycii Fructus* 15g, *Ligustri Lucidi Fructus* 15g, *Poria* 10g, *Ophiopogonis Radix* 10g, *Hedyotis Diffusae Herba* 30g, *Curcumae Rhizoma* 15g, *Glycyrrhizae Radix et Rhizoma* 6g.

^m^  *Hedyotis Diffusae Herba* 15g, *Sophorae Flos* 15g, *Scutellariae Barbatae Herba* 15g, *Psoraleae Fructus* 10g, *Phellodendri Chinensis Cortex* 10g, *Rehmanniae Radix* 15g, *Myristicae Semen* 10g, *Lablab Semen Album* 15g, ***Coicis Semen* 20g,** *Cimicifugae Rhizoma* 10g, *Puerariae Lobatae Radix* 10g, *Poria* 15g, *Paeoniae Radix Rubra* 10g, *Paeoniae Radix Alba* 15g, *Dioscoreae Rhizoma* 15g, *Ganoderma* 15g, *Astragali Radix* 30g, *Salviae Miltiorrhizae Radix et Rhizoma* 15g, *Pseudostellariae Radix* 15g.

^n^  *Astragali Radix*, *Scutellariae Barbatae Herba*, *Hedyotis Diffusae Herba*, *Codonopsis Radix*, *Atractylodis Macrocephalae Rhizoma*, *Poria*, *Aurantii Fructus*, *Magnoliae Officinalis Cortex*, *Mume Flos*, *Polygoni Cuspidati Rhizoma et Radix*, ***Coicis Semen*,** *Amomi Fructus*, *Setariae Fructus Germinatus* (dosage information is not available).

^o^  *Astragali Radix* 30g, *Codonopsis Radix* 15g, *Atractylodis Macrocephalae Rhizoma* 15g, *Ligustri Lucidi Fructus* 15g, *Lycii Fructus* 10g, *Cuscutae Semen* 10g, *Curcumae Rhizoma* 15g, ***Coicis Semen* 30g,** *Smilacis Glabrae Rhizoma* 15g, *Gekko Japonicus Dumeril et Bibron* 10g, *Paridis Rhizoma* 10g, *Scutellariae Barbatae Herba* 30g, *Cyathulae Radix* 15g, *Glycyrrhizae Radix et Rhizoma* 5g.

^p^  *Astragali Radix* 30g, ***Coicis Semen* 30g,** *Scutellariae Barbatae Herba* 30g, *Atractylodis Macrocephalae Rhizoma* 15g, *Codonopsis Radix* 15g, *Curcumae Rhizoma* 15g, *Ligustri Lucidi Fructus* 15g, *Cyathulae Radix* 15g, *Smilacis Glabrae Rhizoma* 15g, *Gekko Japonicus Dumeril et Bibron* 10g, *Lycii Fructus* 10g, *Paridis Rhizoma* 10g, *Cuscutae Semen* 10g, *Glycyrrhizae Radix et Rhizoma* 5g.

^q^  ***Coicis Semen* 30g,** *Astragali Radix* 30g, *Pseudostellariae Radix* 20g, *Lycii Fructus* 15g, *Ligustri Lucidi Fructus* 15g, *Salviae Miltiorrhizae Radix et Rhizoma* 10g, *Cyathulae Radix* 6g, *Citri Reticulatae Pericarpium* 6g, *Atractylodis Macrocephalae Rhizoma* 6g.

^r^  *Bletillae Rhizoma* 10g, *Typhae Pollen* 10g, *Angelicae Sinensis Radix* 15g, ***Coicis Semen* 20g.**

^s^  *Pseudostellariae Radix* 15g, *Salviae Miltiorrhizae Radix et Rhizoma* 15g, *Astragali Radix* 30g, *Ganoderma* 15g, *Dioscoreae Rhizoma* 15g, *Paeoniae Radix Alba* 15g, *Paeoniae Radix Rubra* 10g, *Poria* 15g, *Puerariae Lobatae Radix* 10g, *Cimicifugae Rhizoma* 10g, ***Coicis Semen* 20g,** *Lablab Semen Album* 15g, *Myristicae Semen* 10g, *Rehmanniae Radix* 15g, *Phellodendri Chinensis Cortex* 10g, *Psoraleae Fructus* 10g, *Scutellariae Barbatae Herba* 15g, *Sophorae Flos* 15g, *Hedyotis Diffusae Herba* 15g.

^t^  *Houttuyniae Herba* 15g, *Astragali Radix* 12g, *Codonopsis Radix* 12g, *Atractylodis Macrocephalae Rhizoma* 8g, *Glycyrrhizae Radix et Rhizoma* 5g, ***Coicis Semen* 10g.**

^u^  *Astragali Radix* 30g, ***Coicis Semen* 20g**, *Paeoniae Radix Alba* 20g, *Poria* 20g, *Scutellariae Radix* 15g, *Curaumae Radix* 15g, *Herba Patriniae* 15g, *Plantaginis Herba* 15g, *Curcumae Rhizoma* 15g, *Codonopsis Radix* 15g, *Atractylodis Macrocephalae Rhizoma* 15g, *Galli Gigerii Endothelium Corneum* 15g, *Coptidis Rhizoma* 10g, *Angelicae Sinensis Radix* 10g, *Bupleuri Radix* 10g, *Glycyrrhizae Radix et Rhizoma* 6g.

^v^  *Astragali Radix* 30g, *Pseudostellariae Radix* 15g, ***Coicis Semen* 20g,** *Atractylodis Macrocephalae Rhizoma*15g, *Phellodendri Chinensis Cortex* 10g, *Scutellariae Radix* 10g, *Cimicifugae Rhizoma* 10g, *Puerariae Lobatae Radix* 10g, *Psoraleae Fructus* 10g, *Lobeliae Chinensis Herba* 15g, *Scutellariae Barbatae Herba* 15g, *Hedyotis Diffusae Herba* 15g, *Rehmanniae Radix* 15g, *Dioscoreae Rhizoma* 15g, *Lablab Semen Album* 15g, *Pulsatillae Radix* 10g, *Myristicae Semen* 10g, *Poria* 15g, *Paeoniae Radix Rubra* 10g.

^w^  *Sophorae Flavescentis Radix*, ***Coicis Semen***, *Sparganii Rhizoma*, *Curcumae Rhizoma*, *Pseudobulbus Cremastrae seu Pleiones* (dosage information is not available).

^x^  *Scutellariae Barbatae Herba* 30g, *Hedyotis Diffusae Herba* 30g, *Astragali Radix* 15g, *Atractylodis Macrocephalae Rhizoma* 15g, *Poria* 15g, *Mume Fructus* 15g, ***Coicis Semen* 15g,** *Sanguisorbae Radix* 12g, *Typhae Pollen* 12g, *Paeoniae Radix Alba* 12g, *Pseudostellariae Radix* 10g, *Massa Dermentata Medicinalis* 10g, *Puerariae Lobatae Radix* 10g, *Scutellariae Radix* 10g, *Granati Pericarpium* 10g, *Saposhnikoviae Radix* 10g, *Notoginseng Radix et Rhizoma* 6g, *Glycyrrhizae Radix et Rhizoma* 5g, *Ligustri Lucidi Fructus* 2g.

^y^  *Herba Patriniae* 30g, *Lonicerae Japonicae Flos* 20g, ***Coicis Semen* 20g,** *Forsythiae Fructus* 20g,

*Sophorae Flavescentis Radix* 20g, *Phellodendri Chinensis Cortex* 15g, *Atractylodis Rhizoma* 15g, *Atractylodis Macrocephalae Rhizoma* 15g, *Coptidis Rhizoma* 10g.

^z^  *Herba Patriniae* 30g, ***Coicis Semen* 20g,** *Lonicerae Japonicae Flos* 20g, *Phellodendri Chinensis Cortex* 15g, *Sophorae Fructus* 15g, *Aurantii Fructus* 15g, *Sanguisorbae Radix* 15g, *Dryopteridis Crassirhizomatis Rhizoma* 15g, *Atractylodis Rhizoma* 15g, *Atractylodis Macrocephalae Rhizoma* 10g, *Coptidis Rhizoma* 10g.

^aa^  *Lonicerae Japonicae Flos* 20g, *Forsythiae Fructus* 20g, *Herba Patriniae* 20g, ***Coicis Semen* 20g,** *Sophorae Flavescentis Radix* 15g, *Phellodendri Chinensis Cortex* 15g, *Atractylodis Rhizoma* 15g, *Atractylodis Macrocephalae Rhizoma* 15g, *Coptidis Rhizoma* 10g, *Glycyrrhizae Radix et Rhizoma* 10g.

^ab^  *Glycyrrhizae Radix et Rhizoma* 10g, *Coptidis Rhizoma* 6g, *Scutellariae Radix* 10g, *Mume Fructus* 15g, *Herba Patriniae* 30g, *Lonicerae Japonicae Flos* 20g, *Sophorae Flos* 15g, *Dioscoreae Rhizoma* 15g, *Sanguisorbae Radix* 30g, ***Coicis Semen* 30g,** *Poria* 15g, *Puerariae Lobatae Radix* 15g, *Scrophulariae Radix* 15g, *Atractylodis Macrocephalae Rhizoma* 15g, *Pseudostellariae Radix* 15g, *Astragali Radix* 30g.

^ac^  *Lonicerae Japonicae Flos* 20g, *Forsythiae Fructus* 20g, *Herba Patriniae* 20g, ***Coicis Semen* 30g,** *Sophorae Flavescentis Radix* 15g, *Phellodendri Chinensis Cortex* 15g, *Atractylodis Rhizoma* 15g, *Atractylodis Macrocephalae Rhizoma* 15g, *Coptidis Rhizoma* 10g, *Glycyrrhizae Radix et Rhizoma* 10g.

^ad^  *Astragali Radix* 30g, *Pseudostellariae Radix* 15g, *Atractylodis Macrocephalae Rhizoma* 15g, *Scrophulariae Radix* 15g, *Puerariae Lobatae Radix* 15g, *Poria* 15g, ***Coicis Semen* 30g,** *Sanguisorbae Radix* 30g, *Dioscoreae Rhizoma* 15g, *Sophorae Flos* 15g, *Lonicerae Japonicae Flos* 20g, *Herba Patriniae* 30g, *Mume Fructus* 15g, *Scutellariae Radix* 10g, *Coptidis Rhizoma* 6g, *Glycyrrhizae Radix et Rhizoma* 10g.

^ae^  *Angelicae Sinensis Radix* 9g, *Rehmanniae Radix* 12g, *Sophorae Flos* 12g, *Ostreae Concha* 15g, *Agrimoniae Herba* 30g, *Sanguisorbae Radix* 12g, ***Coicis Semen* 15g,** *Herba Patriniae* 15g, *Aucklandiae Radix* 3g, *Houttuyniae Herba* 15g, *Notoginseng Radix et Rhizoma* 1g.

^af^  *Lonicerae Japonicae Flos* 20g, *Forsythiae Fructus* 15g, *Herba Patriniae* 20g, *Sophorae Flavescentis Radix* 15g, *Phellodendri Chinensis Cortex* 15g, *Atractylodis Rhizoma* 30g, *Atractylodis Macrocephalae Rhizoma* 30g, ***Coicis Semen* 30g,** *Lysimachiae Herba* 30g, *Plantaginis Herba* 30g, *Agrimoniae Herba* 30g, *Linderae Radix* 15g, *Hordei Fructus Germinatus* 30g.

^ag^  *Mume Fructus* 15g, *Codonopsis Radix* 20g, *Sophorae Flos* 15g, *Astragali Radix* 30g, *Sanguisorbae Radix* 30g, *Atractylodis Macrocephalae Rhizoma* 15g, *Puerariae Lobatae Radix* 15g, *Poria* 20g, *Herba Patriniae* 30g, ***Coicis Semen* 30g,** *Smilacis Glabrae Rhizoma* 30g, *Lonicerae Japonicae Flos* 20g, *Dioscoreae Rhizoma* 15g.

^ah^  *Sophorae Flos* 10g, *Sanguisorbae Radix* 10g, *Coptidis Rhizoma* 5g, *Herba Patriniae* 12g, *Pulsatillae Radix* 15g, *Lobeliae Chinensis Herba* 10g, *Fraxini Cortex* 10g, ***Coicis Semen* 20g,** *Hedyotis Diffusae Herba* 10g, *Aucklandiae Radix* 10g, *Bletillae Rhizoma* 10g, *Notoginseng Radix et Rhizoma* 10g, *Glycyrrhizae Radix et Rhizoma* 4g.

^ai^  *Astragali Radix* 30g, *Codonopsis Radix* 12g, *Rehmanniae Radix* 15g, *Ophiopogonis Radix* 30g, *Glehniae Radix* 12g, ***Coicis Semen* 15g,** *Notoginseng Radix et Rhizoma* 6g, *Pulsatillae Radix* 15g, *Mume Fructus* 15g, *Halloysitum Rubrum* 15g.

^aj^  *Codonopsis Radix* 20g, *Astragali Radix* 30g, *Atractylodis Macrocephalae Rhizoma* 15g, *Poria* 20g, ***Coicis Semen* 30g,** *Dioscoreae Rhizoma* 15g, *Lonicerae Japonicae Flos* 20g, *Smilacis Glabrae Rhizoma* 30g, *Herba Patriniae* 30g, *Puerariae Lobatae Radix* 15g, *Sanguisorbae Radix* 30g, *Sophorae Flos* 15g, *Mume Fructus* 15g, *Glycyrrhizae Radix et Rhizoma* 10g.

^ak^  *Pseudostellariae Radix* 15g, *Astragali Radix* 30g, *Atractylodis Macrocephalae Rhizoma* 15g, *Poria* 15g, ***Coicis Semen* 30g,** *Dioscoreae Rhizoma* 15g, *Lonicerae Japonicae Flos* 20g, *Scutellariae Radix* 10g, *Coptidis Rhizoma* 6g, *Herba Patriniae* 30g, *Scrophulariae Radix* 15g, *Puerariae Lobatae Radix* 15g, *Sanguisorbae Radix* 30g, *Sophorae Flos* 15g, *Mume Fructus* 15g, *Glycyrrhizae Radix et Rhizoma* 10g.

^al^  *Angelicae Sinensis Radix* 9g, *Rehmanniae Radix* 12g, *Sophorae Flos* 12g, *Ostreae Concha* 15g, *Agrimoniae Herba* 30g, *Sanguisorbae Radix* 12g, ***Coicis Semen* 15g,** *Herba Patriniae* 15g, *Pulsatillae Radix* 15g, *Aucklandiae Radix* 9g.

^am^  *Astragali Radix* 30g, *Atractylodis Macrocephalae Rhizoma* 12g, *Dioscoreae Rhizoma* 12g, *Sophorae Flos* 10g, *Platycladi Cacumen* 10g, *Puerariae Lobatae Radix* 15g, *Saposhnikoviae Radix* 10g, ***Coicis Semen* 20g,** *Chebulae Fructus* 10g, *Aucklandiae Radix* 6g, *Paeoniae Radix Rubra* 15g, *Paeoniae Radix Alba* 15g, *Portulacae Herba* 20g, *Herba Patriniae* 20g, *Pulsatillae Radix* 15g, *Crataegi Fructus* 15g, *Glycyrrhizae Radix et Rhizoma* 6g.

^an^  *Angelicae Sinensis Radix* 9g, *Rehmanniae Radix* 12g, *Sophorae Flos* 12g, *Ostreae Concha* 15g, *Agrimoniae Herba* 30g, *Sanguisorbae Radix* 12g, ***Coicis Semen* 15g,** *Herba Patriniae* 15g, *Pulsatillae Radix* 15g, *Aucklandiae Radix* 9g.

^ao^  *Astragali Radix* 30g, *Poria* 20g, *Paeoniae Radix Alba* 20g, ***Coicis Semen* 20g,** *Codonopsis Radix* 15g, *Atractylodis Macrocephalae Rhizoma* 15g, *Scutellariae Radix* 15g, *Plantaginis Herba* 15g, *Herba Patriniae* 15g, *Curcumae Rhizoma* 15g, *Curcumae Radix* 15g, *Galli Gigerii Endothelium Corneum* 15g, *Bupleuri Radix* 10g, *Coptidis Rhizoma* 10g, *Angelicae Sinensis Radix* 10g, *Glycyrrhizae Radix et Rhizoma* 6g.

^ap^  *Astragali Radix* 30g, *Codonopsis Radix* 30g, *Poria* 30g, *Dioscoreae Rhizoma* 30g, *Hedyotis Diffusae Herba* 30g, *Paridis Rhizoma* 30g, *Scutellariae Barbatae Herba* 30g, *Atractylodis Macrocephalae Rhizoma* 10g, ***Coicis Semen* 50g,** *Citri Reticulatae Pericarpium* 5g, *Coptidis Rhizoma* 3g, *Pogostemonis Herba* 15g, *Eupatorii Herba* 15g.

^aq^  *Angelicae Sinensis Radix* 10g, *Paeoniae Radix Alba* 10g, *Persicae Semen* 10g, *Alismatis Rhizoma* 10g, *Stephaniae Tetrandrae Radix* 10g, *Trachelospermi Caulisetfolium* 10g, *Cyathulae Radix* 15g, *Achyranthis Bidentatae Radix* 15g, ***Coicis Semen* 15g,** *Taraxaci Herba* 15g, *Lonicerae Japonicae Flos* 15g, *Squama Manis* 5g, *Ostreae Concha* 30g, *Glycyrrhizae Radix et Rhizoma* 6g.

^ar^  *Chaenomelis Fructus* 15g, *Mori Ramulus* 50g, *Achyranthis Bidentatae Radix* 15g, *Spatholobi Caulis* 25g, *Luffae Fructus Retinervus* 10g, *Trachelospermi Caulisetfolium* 15g, *Liquidambaris Fructus* 20g, *Stephaniae Tetrandrae Radix* 15g, ***Coicis Semen* 20g,** *Solanum Nigrum* 25g, *Alismatis Rhizoma* 15g, *Lonice Raejaponicae Caulis* 25g, *Persicae Semen* 15g, *Paeoniae Radix Rubra* 15g, *Squama Manis* 15g, *Angelicae Sinensis Radix* 20g.

^as^  *Spatholobi Caulis* 24g, *Sargentodoxae Caulis* 24g, *Violae Herba* 24g, *Herba Patriniae* 24g, *Taraxaci Herba* 24g, *Hedyotis Diffusae Herba* 24g, ***Coicis Semen* 24g,** *Smilacis Glabrae Rhizoma* 24g, *Scutellariae Barbatae Herba* 10g, *Cyperi Rhizoma* 10g, *Sparganii Rhizoma* 10g, *Leonuri Herba* 10g, *Curcumae Rhizoma* 10g, *Aucklandiae Radix* 10g.

^at^  *Persicae Semen* 10g, *Paeoniae Radix Rubra* 10g, *Angelicae Sinensis Radix* 10g, *Liquidambaris Fructus* 10g, *Cyathulae Radix* 15g, ***Coicis Semen* 15g,** *Smilacis Glabrae Rhizoma* 15g, *Solanum Nigrum* 15g, *Taraxaci Herba* 15g, *Lonicerae Japonicae Flos* 15g, *Squama Manis* 5g, *Ostreae Concha* 30g, *Glycyrrhizae Radix et Rhizoma* 9g.

^au^  ***Coicis Semen* 30g,** *Ligustri Lucidi Fructus* 10g, *Taxilli Herba* 10g, *Polyporus Umbellatus* 10g, *Astragali Radix* 30g, *Cinnamomi Ramulus* 10g, *Achyranthis Bidentatae Radix* 10g, *Atractylodis Macrocephalae Rhizoma* 10g, *Polygoni Avicularis Herba* 10g, *Dioscoreae Rhizoma* 10g, *Alismatis Rhizoma* 10g, *Eucommiae Cortex* 10g, *Ginseng Radix et Rhizoma* 10g, *Dianthi Herba* 10g, *Poria* 10g, *Lycopi Herba* 10g, *Pyrrosiae Folium* 15g.

^av^  *Codonopsis Radix* 15g, ***Coicis Semen* 30g,** *Astragali Radix* 30g, *Poria* 10g, *Alismatis Rhizoma* 15g,

*Lycopi Herba* 10g, *Atractylodis Macrocephalae Rhizoma* 15g, *Citri Reticulatae Pericarpium* 10g, *Cinnamomi Ramulus* 10g.

^aw^  ***Coicis Semen* 30g,** *Astragali Radix* 30g, *Pyrrosiae Folium* 15g, *Ginseng Radix* *et Rhizoma* 10g, *Atractylodis Macrocephalae Rhizoma* 10g, *Poria* 10g, *Cinnamomi Ramulus* 10g, *Dioscoreae Rhizoma* 10g, *Taxilli Herba* 10g, *Eucommiae Cortex* 10g, *Achyranthis Bidentatae Radix* 10g, *Ligustri Lucidi Fructus* 10g, *Dianthi Herba* 10g, *Polygoni Avicularis Herba* 10g, *Plantaginis Semen* 10g, *Polyporus* 10g, *Alismatis Rhizoma* 10g, *Lycopi Herba* 10g.

^ax^  *Aucklandiae Radix* 9g, *Arecae Semen* 9g, *Citri Reticulatae Pericarpium* 9g, *Angelicae Sinensis Radix* 9g, *Paeoniae Radix Rubra* 12g, ***Coicis Semen* 30g,** *Lablab Semen Album* 9g, *Radix et Rhizoma Thalictri* 6g, *Chebulae Fructus* 9g, *Glycyrrhizae Radix et Rhizoma* 6g.

^ay^  *Ginseng Radix et Rhizoma* 10g, *Atractylodis Macrocephalae Rhizoma* 15g, *Poria* 15g, *Glycyrrhizae Radix et Rhizoma* 10g, *Lablab Semen Album* 10g, *Dioscoreae Rhizoma* 10g, *Amomi Fructus* 10g, ***Coicis Semen* 10g,** *Platycodonis Radix* 6g, *Jujubae Fructus* 20g.

^az^  *Poria* 30g, *Polyporus Umbellatus* 15g, *Atractylodis Macrocephalae Rhizoma* 12g, *Alismatis Rhizoma* 15g, *Cinnamomi Ramulus* 10g, *Citri Reticulatae Pericarpium* 10g, *Zingiberis Rhizoma Recens* 10g, *Mori Cortex* 10g, *Arecae Pericarpium* 10g, ***Coicis Semen* 12g**, *Liquidambaris Fructus* 10g, *Salviae Miltiorrhizae Radix et Rhizoma* 10g, *Spatholobi Caulis* 30g, *Lycopi Herba* 15g.

^bb^  *Atractylodis Macrocephalae Rhizoma* 20g, *Poria* 20g, *Codonopsis Radix* 20g, ***Coicis Semen* 20g,** *Hedyotis Diffusae Herba* 15g, *Galli Gigerii Endothelium Corneum* 15g, *Glycyrrhizae Radix et Rhizoma* 15g, *Trichosanthis Pericarpium* 10g, *Scutellariae Barbatae Herba* 10g, *Akebia trifoliata* 10g, *Ranunculi Ternati Radix* 10g.

^bc^  *Hedyotis Diffusae Herba* 30g, *Astragali Radix* 30g, ***Coicis Semen* 30g,** *Curcumae Rhizoma* 15g, *Atractylodis Macrocephalae Rhizoma* 15g, *Poria* 15g, *Pseudostellariae Radix* 15g, *Amomi Fructus* 15g, *Citri Reticulatae Pericarpium* 15g, *Platycodonis Radix* 10g, *Lablab Semen Album* 10g, *Alismatis Rhizoma* 10g, *Polyporus Umbellatus* 10g, *Dioscoreae Rhizoma* 10g, *Glycyrrhizae Radix et Rhizoma* 10g.

^bd^  *Astragali Radix* 30g, *Citri Reticulatae Pericarpium* 10g, *Ligustri Lucidi Fructus* 10g, *Lycii Fructus* 10g, *Angelicae Sinensis Radix* 10g, *Paeoniae Radix Alba* 15g, *Poria* 15g, ***Coicis Semen* 30g,** *Galli Gigerii Endothelium Corneum* 10g, *Spatholobi Caulis* 15g, *Agrimoniae Herba* 12g.

^be^  *Astragali Radix* 30g, *Ganoderma* 30g, *Ginseng Radix et Rhizoma* 30g, ***Coicis Semen* 30g,** *Scutellariae Barbatae Herba* 30g, *Poria* 15g, *Atractylodis Macrocephalae Rhizoma* 15g, *Crataegi Fructus* 12g, *Massa Dermentata Medicinalis* 12g, *Hordei Fructus Germinatus* 12g, *Magnoliae Officinalis Cortex* 12g, *Citri Reticulatae Pericarpium* 9g, *Galli Gigerii Endothelium Corneum* 9g, *Gekko japonicus Dumeril et Bibron* 9g.

^bf^  *Astragali Radix* 15g, *Ginseng Radix et Rhizoma* 15g, *Poria* 15g, *Polyporus Umbellatus* 15g, *Lycii Fructus* 15g, *Ligustri Lucidi Fructus* 15g, *Paeoniae Radix Alba* 15g, *Spatholobi Caulis* 15g, *Galli Gigerii Endothelium Corneum* 15g, *Angelicae Sinensis Radix* 12g, *Citri Reticulatae Pericarpium* 12g, *Agrimoniae Herba* 12g, ***Coicis Semen* 30g.**

^bg^  *Codonopsis Radix* 120g, *Astragali Radix* 180g, *Angelicae Sinensis Radix* 60g, *Rehmanniae Radix Praeparata* 60g, ***Coicis Semen* 60g,** *Atractylodis Macrocephalae Rhizoma* 60g, *Scutellariae Barbatae Herba* 60g, *Hedyotis Diffusae Herba* 60g, *Pseudobulbus Cremastrae seu Pleiones* 30g, *Prunellae Spica* 60g, *Curcumae Rhizoma* 30g, *Lobeliae Chinensis Herba* 60g, *Sparganii Rhizoma* 30g, *Glycyrrhizae Radix et Rhizoma* 30g.

^bh^  *Astragali Radix* 30g, *Codonopsis Radix* 15g, *Trichosanthis Radix* 15g, *Atractylodis Macrocephalae Rhizoma* 20g, *Paeoniae Radix Alba* 15g, ***Coicis Semen* 30g,** *Agrimoniae Herba* 30g, *Spatholobi Caulis* 30g, *Polyporus Umbellatus* 15g, *Poria* 15g, *Salviae Miltiorrhizae Radix et Rhizoma* 15g, *Scrophulariae Radix* 10g, *Scutellariae Barbatae Herba* 20g, *Hedyotis Diffusae Herba* 30g.

^bi^  *Aconiti Lateralis Radix Praeparata* 10g, *Zingiberis Rhizoma Recens* 15g, *Astragali Radix* 25g, *Atractylodis Macrocephalae Rhizoma* 15g, *Pinelliae Rhizoma* 8g, *Citri Reticulatae Pericarpium* 15g, *Poria* 20g, *Paeoniae Radix Alba* 15g, *Aurantii Fructus* 15g, *Persicae Semen* 15g, *Leonuri Herba* 30g, ***Coicis Semen* 30g.**

^bj^  *Astragali Radix* 30g, *Atractylodis Macrocephalae Rhizoma* 30g, *Gynostemma Compressum* 30g, ***Coicis Semen* 30g**, *Hedyotis Diffusae Herba* 30g, *Vitis Romanetii Radix* 30g, *Actinidia Arguta* 30g, *Polyporus Umbellatus* 15g, *Dendrobii Herba* 15g, *Fruit of Fiverleaf Akebia* 15g, *Notoginseng Radix et Rhizoma* 15g, *Galli Gigerii Endothelium Corneum* 10g, *Glycyrrhizae Radix et Rhizoma* 10g.

^bk^  *Astragali Radix* 30g, *Hedyotis Diffusae Herba* 30g, ***Coicis Semen* 30g,** *Pseudostellariae Radix* 15g, *Poria* 15g, *Curcumae Rhizoma* 15g, *Atractylodis Macrocephalae Rhizoma* 15g, *Amomi Fructus* 15g, *Citri Reticulatae Pericarpium* 15g, *Platycodonis Radix* 10g, *Polyporus Umbellatus* 10g, *Alismatis Rhizoma* 10g, *Lablab Semen Album* 10g, *Dioscoreae Rhizoma* 10g, *Glycyrrhizae Radix et Rhizoma* 6g.

^bl^  *Dioscoreae Rhizoma* 30g, *Glycyrrhizae Radix et Rhizoma* 20g, *Jujubae Fructus* 15g, ***Coicis Semen* 15g,** *Angelicae Sinensis Radix* 12g, *Rehmanniae Radix Praeparata* 12g, *Atractylodis Macrocephalae Rhizoma* 12g, *Codonopsis Radix* 12g, *Asini Corii Colla* 12g, *Massa Dermentata Medicinalis* 12g, *Chuanxiong Rhizoma* 9g, *Paeoniae Radix Rubra* 9g, *Poria* 9g, *Zingiberis Rhizoma* 6g, *Ophiopogonis Radix* 6g, *Armeniacae Semen Amarum* 6g, *Bupleuri Radix* 6g, *Saposhnikoviae Radix* 6g, *Platycodonis Radix* 6g, *Cinnamomi Ramulus* 6g, *Ampelopsis Radix* 3g.

^bm^  *Lycii Fructus* 15g, *Corni Fructus* 15g, *Eucommiae Cortex* 15g, *Dioscoreae Rhizoma* 15g, *Polygonati Rhizoma* 15g, ***Coicis Semen* 30g,** *Atractylodis Macrocephalae Rhizoma* 15g, *Poria* 15g, *Paeoniae Radix Alba* 10g, *Curcumae Radix* 10g, *Salviae Miltiorrhizae Radix et Rhizoma* 10g, *Hedyotis Diffusae Herba* 15g, *Scutellariae Barbatae Herba* 15g, *Citri Reticulatae Pericarpium* 10g, *Galli Gigerii Endothelium Corneum* 15g, *Crataegi Fructus* 15g, *Massa Dermentata Medicinalis* 15g.

^bn^  *Astragali Radix* 30g, *Atractylodis Macrocephalae Rhizoma* 30g, ***Coicis Semen* 30g**, *Eupolyphaga Steleophaga* 30g, *Hedyotis Diffusae Herba* 30g, *Vitis Viniferae Radix* 30g, *Actinidia Arguta* 30g, *Polyporus Umbellatus* 15g, *Dendrobii Herba* 15g, *Fruit of Fiverleaf Akebia* 15g, *Notoginseng Radix et Rhizoma* 15g, *Galli Gigerii Endothelium Corneum* 10g, *Glycyrrhizae Radix et Rhizoma* 10g.

^bo^  *Astragali Radix* 180g, *Codonopsis Radix* 120g, *Lobeliae Chinensis Herba* 60g, *Prunellae Spica* 60g, *Rehmanniae Radix Praeparata* 60g, *Angelicae Sinensis Radix* 60g, *Hedyotis Diffusae Herba* 60g, *Atractylodis Macrocephalae Rhizoma* 60g, ***Coicis Semen* 60g,** *Scutellariae Barbatae Herba* 60g, *Curcumae Rhizoma* 30g, *Sparganii Rhizoma* 30g, *Pseudobulbus Cremastrae seu Pleiones* 30g, *Glycyrrhizae Radix et Rhizoma* 30g.

^bp^  *Codonopsis Radix* 30g, *Astragali Radix* 30g, *Poria* 10g, *Atractylodis Macrocephalae Rhizoma* 10g, *Galli Gigerii Endothelium Corneum* 30g, *Hordei Fructus Germinatus* 15g, *Dioscoreae Rhizoma* 20g, ***Coicis Semen* 10g,** *Pogostemonis Herba* 10g, *Pinelliae Rhizoma* 10g, *Inulae Flos* 10g, *Ganoderma* 10g, *Salviae Miltiorrhizae Radix et Rhizoma* 10g, *Amomi Fructus* 5g, *Amomi Fructus Rotundus* 5g, *Bombyx Batryticatus* 10g, *Albiziae Cortex* 10g.

^bq^  *Aconiti Lateralis Radix Praeparata* 20g, *Poria* 15g, *Cinnamomi Ramulus* 20g, *Pseudostellariae Radix* 25g, *Atractylodis Macrocephalae Rhizoma* 15g, *Zingiberis Rhizoma Recens* 15g, ***Coicis Semen* 20g,** *Polyporus Umbellatus* 15g, *Alismatis Rhizoma* 15g, *Epimedii Folium* 15g, *Corni Fructus* 20g, *Dioscoreae Rhizoma* 15g, *Paeoniae Radix Alba* 15g, *Citri Reticulatae Pericarpium* 10g, *Jujubae Fructus* 10g, *Glycyrrhizae Radix et Rhizoma* 5g.

^br^  *Pseudostellariae Radix* 30g, *Poriae Cutis* 30g, *Atractylodis Macrocephalae Rhizoma* 15g, *Polyporus* 12g, *Alismatis Rhizoma* 12g, *Sparganii Rhizoma* 12g, *Curcumae Rhizoma* 12g, *Hedyotis Diffusae Herba* 12g, *Scutellariae Barbatae Herba* 12g, *Lycii Fructus* 12g, *Dioscoreae Rhizoma* 20g, ***Coicis Semen* 20g,** *Arecae Pericarpium* 15g, *Glycyrrhizae Radix et Rhizoma* 6g.

^bs^  *Pseudostellariae Radix* 30g, *Poria* 15g, *Atractylodis Macrocephalae Rhizoma* 10g, *Lablab Semen Album* 10g, *Dioscoreae Rhizoma* 30g, *Nelumbinis Semen* 10g, ***Coicis Semen* 30g,** *Citri Reticulatae Pericarpium* 5g, *Platycodonis Radix* 5g, *Jujubae Fructus* 10g, *Glycyrrhizae Radix et Rhizoma* 5g,

*Amomi Fructus* 10g.

^bt^  *Euphorbiae Semen* 6g, *Aquilariae Lignum Resinatum* 10g, *Arecae Semen* 10g, *Phytolaccae Radix* 20g, *Curcumae Rhizoma* 30g, *Carthami Flos* 30g, *Persicae Semen* 40g, ***Coicis Semen* 50g,** *Lysionoti Herba* 50g, *Polyporus* 50g, *Astragali Radix* 80g.

^bu^  *Codonopsis Radix* 15g, *Astragali Radix* 30g, *Lycii Fructus* 10g, *Mori Cortex* 20g, *Atractylodis Macrocephalae Rhizoma* 15g, *Poriae Cutis* 30g, ***Coicis Semen* 12g,** *Scutellariae Barbatae Herba* 30g, *Paridis Rhizoma* 10g, *Ganoderma* 10g, *Hedyotis Diffusae Herba* 30g, *Angelicae Sinensis Radix* 10g, *Pinelliae Rhizoma* 8g, *Spatholobi Caulis* 10g, *Citri Reticulatae Pericarpium* 6g, *Citri Reticulatae Pericarpium Viride* 6g, *Glycyrrhizae Radix et Rhizoma* 6g.

^bv^  *Astragali Radix* 80g, *Polyporus Umbellatus* 50g, *Lysionoti Herba* 50g, *Phytolaccae Radix* 20g, *Euphorbiae Semen* 6g, ***Coicis Semen* 50g,** *Persicae Semen* 40g, *Carthami Flos* 30g, *Curcumae Rhizoma* 30g, *Aquilariae Lignum Resinatum* 10g, *Arecae Semen* 10g.

^bw^  *Pseudostellariae Radix 1*5g, *Poria* 15g, *Atractylodis Macrocephalae Rhizoma* 15g, *Lablab Semen Album* 10g, *Platycodonis Radix* 10g, *Citri Reticulatae Pericarpium* 15g, *Dioscoreae Rhizoma* 10g, *Amomi Fructus* 15g, ***Coicis Semen* 30g,** *Glycyrrhizae Radix et Rhizoma* 10g, *Astragali Radix* 30g, *Polyporus Umbellatus* 10g, *Alismatis Rhizoma* 10g, *Curcumae Rhizoma* 15g, *Hedyotis Diffusae Herba* 30g.

**References**

Chen, J. (2019). Clinical observation on the prevention of radiation proctitis of cervical cancer by traditional Chinese medicine retention enema. China's Naturopathy. 27, 17–19. doi: 10.19621/j.cnki.11-3555/r.2019.0110

Chen, M., Guo, X., Xiong, Y., Chen, X., Pu, C., Xie, Y. (2020). Efficacy and survival of Peiyuan-Guben combined with chemoradiotherapy in patients with advanced cervical cancer. Electron. J. Pract. Gynecol. Endocrinol. 7, 12–13. doi: 10.16484/j.cnki.issn2095-8803.2020.31.009

Chen, X., Chen, C., Sun, Y., Zhang, H., Gao, C., Chen, S. (2018). Efficacy of Yishen Kangma decoction combined with IL-2 intraperitoneal infusion in the treatment of ovarian cancer related ascites. Chin. J. Clin. Pharmacol. Ther. 23, 1408–1414.

Chen, Z., Ren, H., Peng, T. (2014). Clinical effect of Fuzheng Xiaooma decoction combined with paclitaxel in the treatment of advanced ovarian cancer. Anti-Tumor Pharmacy. 4, 226–228.

Chen, Z., Tan, J., Yu, M. (2014). Treatment of 50 cases of cervical cancer with Fuzheng Jiedu Decoction combined with intensity-modulated radiotherapy. Chin. Med. Mod. Distance Educ. China. 12, 40–42.

Dai, J., Li, J., Yang, S., Liu, L., Yang, B., Zhao, J., et al. (2015). Study on the treatment of lymph fluid exudation and prevention of lymphatic cyst formation after pelvic lymph node dissection for cervical cancer by external application of traditional Chinese medicine and oral administration. J. Clin. Exp. Med. 14, 1504–1507.

Dong, J., Zhao, X., Shu, J. (2012). Clinical observation on the prevention of radiation proctitis of cervical cancer by traditional Chinese medicine retention enema. J. Mod. Oncol. 20,1421-1423.

Dong, S., Liang, H., Li, Y. (2011). Clinical observation of Yiqi Yangyin enema decoction in the treatment of advanced radiation proctitis of cervical cancer. Clin. J. Tradit. Chin. Med. 23, 944–945. doi: 10.16448/j.cjtcm.2011.11.002

Du, Y., Jia, N., Xie, P., Xia, S., Song, X. (2018). Curative effect of Qingre Lishi Jiedu decoction and Western medicine enema on radiation enteritis caused by radiotherapy for cervical cancer. J. Prev. Med. Chin. P.L.A. 36, 1588–1591.

Fan, S., Zheng, J. (2021). Efficacy of Yiqi Huoxue recipe in the treatment of advanced cervical cancer and its influence on serum tumor markers. Matern. Child Health Care China. 36, 1226–1229. doi: 10.19829/j.zgfybj.issn.1001-4411.2021.06.003

Feng, B., Feng, Y., Shi, X. (2019). Cisplatin concurrent radiotherapy combined with Fuzhengquxie traditional Chinese medicine in the treatment of advanced cervical cancer. Mod. J. Int. Tradit. Chin. Western Med. 28, 2247–2249+2253.

Fu, Y., Lei, C. (2000). 28 cases of pelvic lymphocysts after radical resection of cervical cancer treated by internal and external application of Chinese medicine. Shanxi J. Tradit. Chin. Med. 10.

Gong, S., You, J., Ren, C. (2018). Clinical effect of self-made Fuzhengyin No.1 recipe combined with TP chemotherapy in the treatment of cervical cancer. Acta Chin. Med. Pharmacol. 46, 98–100. doi: 10.19664/j.cnki.1002-2392.180024

Guo, X., Peng, W., Wang, C. (2012). Clinical study of Fuzheng Jiedu decoction combined with intensity-modulated radiotherapy in the treatment of cervical cancer. Lishizhen Medicine and Materia Medica Research. 23, 775–776.

Han, L., Lu, Y., Xie, Q., Wang, J. (2013). Effect of microwave combined with traditional Chinese medicine enema on pelvic lymphatic cyst after cervical cancer surgery. J. Chin. Oncol. 19, 22–24.

Hao, Y. (2011). Clinical Study on Attenuated Effect of Modified Shenlingbaizhu Powder on Postoperative Chemotherapy of Ovarian Cancer. Liaoning University of Traditional Chinese Medicine, Shenyang, China.

He, M., Deng, K., Li, S., Ran, W., Zhang, Y. (2021). Clinical effects of Modified Changpi Decoction combined with conventional treatment on patients with radiation enteritis caused by radiotherapy of cervical cancer. Chin. Tradit. Patent Med. 43, 2694–2698.

Hu, Y., Huang, A. (2019). Efficacy of Changfeng Guyu decoction assisted by Western medicine enema in the treatment of acute radiation proctitis caused by radiotherapy for cervical cancer. J Emerg. Tradit. Chin. Med. 28:1624–1627.

Hu, Y., Liu, Y., Wu, C., Chen, C., Wang, Y., Li, X., et al. (2005). Clinical prevention and treatment effect of ShenlingBaizhu Powder on radiation injury of rectum. Chin. J. Radiat. Oncol. 304–306.

Huang, J., Li, G., Kong, X., Wang, L., Zhao, M., Long, Q. (2014). Effect of Fuzheng Zhuoma decoction on the expression of CD68 and CD83 in peripheral blood of patients with cervical cancer. Mod. Diag. Treat. 25, 2442–2444.

Huang, L., Zhai, G., Yang, H. (2020). Effect of modified ShenlingBaizhu Powder on serum inflammatory factors and sex hormones in patients with ovarian cancer after operation. Health Med. Res. Pract. 17, 59–62.

Huang, X., Lv, W., Zhang, M. (2018). Therapeutic effect of combined Chinese and Western medicine on radiation-induced proctitis in patients with cervical cancer radiotherapy. Pract. J. Cardiac Cereb. Pneumal Vasc. Dis. 26, 223–225.

Jian, X., Jiang, Y., Zeng, P. (2015). Clinical observation on 21 cases of advanced cervical cancer treated with Fuzhengxiao decoction plus or minus chemotherapy. Hunan J. Tradit. Chin. Med. 31, 1–3. doi: 10.16808/j.cnki.issn1003-7705.2015.06.001

Jin, Q., Zhao, Y., Ouyang, Y., Li, Q, Wu, G., Yang, B., et al. (2011). Clinical observation of 30 cases of ovarian cancer with ascites treated by external application of traditional Chinese medicine combined with intraperitoneal infusion chemotherapy. Guiding J. Tradit. Chin. Med. Pharm. 17, 30–32. doi: 10.13862/j.cnki.cn43-1446/r.2011.09.060

Jin, W., Kong, C. (2015). Forty-three cases of advanced ovarian cancer treated with Fuzheng Xiaoliu Decoction in combination with paclitaxel. Henan Tradit. Chin. Med. 35:3122–3123. doi: 10.16367/j.issn.1003-5028.2015.12.1343

Lan, X. (2018). Effect of ginseng Fuzheng Guben prescription combined with chemotherapy in the treatment of cervical cancer and its influence on the quality of life of patients. Chin. J. Tradit. Med. Sci. Technol. 25, 95–96.

Li, J., Li, D., Liang, L. (2014). Sixty-six cases of ovarian cancer treated with Shenqi Fuzheng Bandu pill combined with chemotherapy. Chin. Med. Mod. Distance Educ. China. 12, 36.

Li, J., Zhang, B., Guo, Y., Tian, N., Wang, G., Yang, G., et al. (2020). Efficacy of Huangqi Taohong decoction combined with TP Chemotherapy on advanced cervical cancer and its influence on PD-L1 /PD-1 pathway, tumor markers, Th cytokines. Hebei J. Tradit. Chin. Med. 42, 1670–1675.

Li, L., Su, D. (2015). Effect of Fuzheng Peiben decoction on adverse reactions, immune function and quality of life in patients with cervical cancer. J. Hebei Tradit. Chin. Med. Pharmacol. 30, 24–26. doi: 10.16370/j.cnki.13-1214/r.2015.03.008

Li, M., Zhang, Y., Meng, Q. (2006). 48 cases of ovarian cancer were treated with traditional Chinese medicine combined with Western medicine. J. Tradit. Chin. Med. 123–124

Li, Y. (2018). Clinical study on integrated traditional and Western medicine in the treatment for recurrent ovarian cancer stage Ⅲc. Capital Food Med. 25, 15.

Liang, H., Wang, Y., Wang, Y., Li, X. (2015). Treatment of 9 cases of refractory urinary retention after cervical cancer surgery with Chinese medicine combined with acupuncture and microwave permeation. J. Anhui Univ. Chin. Med. 34, 46–49.

Liang, M. (2017). Clinical observation on 48 cases of advanced cervical cancer treated with Fuzheng Jiedu Decoction adjuvant chemoradiotherapy. Chin. J. Tradit. Med. Sci. Technol. 24, 352–354.

Liu, G. (2016). Clinical effect of Chinese medicine combined with acupuncture on urinary retention after senile early cervical cancer surgery. Chin. J. Geriatr. Care. 14, 39–41.

Liu, W., Liu, J., Zheng, L. (2015). Clinical analysis of radiation enteritis after radiotherapy for cervical cancer treated with traditional Chinese medicine. Inner Mongolia J. Tradit. Chin. Medicine. 34, 17. doi: 10.16040/j.cnki.cn15-1101.2015.12.019

Liu, Y., Yang, S., Jin, Q., Liu, H. (2004). Clinical observation on 31 cases of advanced cervical cancer treated by heating and lavage of Qinggong fluid and external irradiation. Guiding J. Tradit. Chin. Med. Pharm. 24–25. doi: 10.13862/j.cnki.cn43-1446/r.2004.04.013

Ma, W. (2021). Clinical effects and adverse reaction analysis of the Fuzheng Jiedu decoction on cervical cancer. Clin. J. Chin. Med. 13, 116–118.

Ma, X., Zhang, X., Liu, H., Liu, S., Zhao, Z. (2011). Clinical study on Tiaogan Jianpi Jiedu Decoction in the prevention and treatment of acute rectal radiation reaction caused by pelvic radiotherapy for cervical cancer. Clin. J. Tradit. Chin. Med.. 23, 946–947. doi: 10.16448/j.cjtcm.2011.11.003

Meng, Y., Liu, K. (2015). Clinical observation of Kangfuxin liquid oral combined with traditional Chinese medicine oral enema in the treatment of acute radiation proctitis after radiotherapy for cervical cancer. J. Emerg. Tradit. Chin. Med. 24, 2213–2215.

Pan, J. (2018). Yiqi Jianpi Yangxue decoction combined with docetaxel and cisplatin in treatment of advanced ovarian cancer. Acta Chin. Med. 33, 1186–1189. doi: 10.16368/j.issn.1674-8999.2018.07.281

Qi, W., Duan, X. (2014). Clinical study on pelvic lymphocysts after cervical cancer surgery with integrated traditional Chinese and Western medicine. Acta Chin. Med. 29, 1105–1106. doi: 10.16368/j.issn.1674-8999.2014.08.055

Qiang, H., Tang, J., Zhao, R., Li, X. (2018). Effect of Zhenwu decoction combined with paclitaxel neoadjuvant chemotherapy on the malignant degree of cervical cancer lesions. J. Hainan Med. Univ. 24, 861–864. doi: 10.13210/j.cnki.jhmu.20180327.002

Qin, L. (2013). Clinical observation and toxicity analysis of postoperative concurrent chemoradiotherapy for cervical cancer combined with oral administration of traditional Chinese medicine. Global Tradit. Chin. Med. 6, 424–426.

Song, Z., Zhang, J., Zhang, H., Sun, X., Zhang, A. (1998). Curative effect of Smecta and Huazhi decoction on diarrhea caused by radiotherapy for cervical cancer. Mod. J. Integr. Tradit. Chin. Western Med. 584-585.

Sun, Z. (2016). Clinical effect of external application of traditional Chinese medicine combined with hyperthermic intraperitoneal chemotherapy on ovarian cancer ascites. China Pract Med. 11, 186–187. doi: 10.14163/j.cnki.11-5547/r.2016.12.136

Tang, Y. (2019). Study on the Adjuvant Effect of Dioscorea Pills During Postoperative Chemotherapy for Epithelial Ovarian Cancer. Qingdao University, Qingdao, China. doi: 10.27262/d.cnki.gqdau.2019.001432

Wang, H., He, F., Zhu, H., Li, X., Sun, K., Li, X., et al. (2021). Effect of Wuling Powder combined with Wupi Decoction for internal and external use combined with free hand massage and external application of traditional Chinese medicine in the treatment of lower limb lymphedema after radical operation of gynecological cancer. China Med. Herald. 18, 113–116+124.

Wang, L., Liu, F., Hao, H., Xue, R., Wang, B. (2018). Effect of transcutaneous electrical acupoint stimulation combined with Shenlingbaizhu Powder on gastrointestinal function and serum inflammatory factor levels after radical operation of ovarian cancer under general anesthesia. Mod. J. Integr. Tradit. Chin. Western Med. 27, 2211–2214.

Wang, L., Yang, J. (2017). Effect of autoimmune cells combined with Xiaoshi cancer suppressor prescription on serum SCC-AG, CyFRA21-1, Bcl-2, Survivin, DcR3 and CEA levels in patients with advanced cervical cancer. Shaanxi J. Tradit. Chin. Med. 38, 821–822.

Wang, Q., Ye, P. (2009). Qinghua-guchang Decoction prevents radiation proctitis in 45 patients with cervical cancer. Tradit. Chin. Medic. Res. 22, 29–31.

Wang, S., Gao, Z. (2015). The short-term clinical effect of Zhenwu decoction combined with seroxanol and carboplatin (TC) in the treatment of patients with ovarian cancer after surgery. Chin. J. Biochem. Pharm. 92–93+96.

Wang, W., Wang, L., Wu, C. (2014). Clinical observation of 30 cases of radiation proctitis of cervical cancer treated by Chinese medicine enema. Chin. J. Ethnomed. Ethnopharm. 23, 95.

Wang, X. (2020). Clinical Study of Yishen Huayu Xiao Decoction Combined with Albumin Binding Paclitaxel+Platinum in the Treatment of Advanced Ovarian Cancer. Nanjing University of Traditional Chinese Medicine, Nanjing, China. doi: 10.27253/d.cnki.gnjzu.2020.000360

Wang, Y., Wang, X., Li, Y. (2014). Clinical observation of Changfukang Prescription in the treatment of 32 cases of radiation enteritis with dampness-heat accumulation after radiotherapy for cervical cancer. Guiding J. Tradit. Chin. Med. Pharm. 20, 29–32. doi: 10.13862/j.cnki.cn43-1446/r.2014.09.010

Xia, Q., Liu, H. (1999). 27 cases of advanced ovarian cancer treated with Chinese medicine and intraperitoneal chemotherapy. Jiangsu J. Tradit. Chin. Med. 26–27.

Xuan, B., Ge, D. (2011). Treating 21 cases of advanced ovarian cancer with Yiqi Jianpi Yangxue decoction combined with chemotherapy. Zhejiang J. Tradit. Chin. Med. 46, 35.

Yang, C., Chang, H., Meng, Y. (2017). Protective mechanism of Shenlingbaizhu Powder on gastrointestinal function during chemotherapy for cervical cancer. Guiding J. oTradit. Chin. Med. Pharm. 23, 121–122+126. doi: 10.13862/j.cnki.cn43-1446/r.2017.23.038

Yang, G., Wang, D., Pang, R., Sun, Y., Xie, D. (2017). Clinical effect evaluation of TCM multi-approach comprehensive therapy for urinary retention after cervical cancer surgery. Progress Mod. Biomed. 17, 5900–5903+6000. doi: 10.13241/j.cnki.pmb.2017.30.023.

Yang, Y. (2016). Clinical efficacy and safety of Fuzheng Jiedu decoction combined with intensity modulated radiotherapy for cervical cancer. Chin. Arch. Tradit. Chin. Med. 34, 2195–2197. doi: 10.13193/j.issn.1673-7717.2016.09.042

Yang, Z., Jiang, Q., Xiao, S., Li, J. (2019). Clinical effective observation of Yichangcuyu decoction combined with western medicine in the treatment of patients with radiation enteritis secondary to radiotherapy for cervical cancer. J. Mod. Oncol. 27, 464–467.

Yi, L., Li, S., He, D. (2017). Clinical observation of oral use of modified Shenling Baizhu powder combined with intraperitoneal perfusion of interleukin-2 for treatment of ascites of Ooarian cancer patients. J. Guangzhou Univ. Tradit. Chin. Med. 34, 31–34. doi: 10.13359/j.cnki.gzxbtcm.2017.01.008

Yin, Q., Huang, Y. (2015). Effect of Fuzhengxiao decoction plus or minus chemotherapy on cervical cancer. Clin. J. Chin. Med. 7, 94–95.

Yu, H., Wang, Z., Xu, R., Liu, L. (2000). Clinical observation on 200 cases of ovarian cancer treated with Shenqi Fuzheng Pudu Pill combined with chemotherapy. Shandong J. Tradit. Chin. Med. 592–593.

Yuan, X., Chen, S. (2012). Summary of 33 cases of ovarian cancer recurrent with ascites after operation treated with integrated traditional Chinese and Western medicine. Hunan J. Tradit. Chin. Med. 28, 20–21. doi: 10.16808/j.cnki.issn1003-7705.2012.01.010

Zhang, C., Zeng, S., Wang, L. (2018). Effect of integrated traditional Chinese and Western medicine on immunological indexes and survival rate of patients with advanced ovarian cancer. Guangming J. Chin. Med. 33, 2248–2250.

Zhang, F., Cui, Y. (2010). Clinical observation on treatment of acute radiation proctitis of cervical cancer with oral Chinese medicine and enema. Liaoning J. Tradit. Chin. Med. 37, 1750–1752. doi: 10.13192/j.ljtcm.2010.09.122.zhangfl.100

Zhang, H. (2010). Clinical observation on treatment of cervical cancer chronic radiation proctitis by traditional Chinese medicine enema. Global Tradit. Chin. Med. 3, 280–282.

Zhang, L. (2020). Clinical effect analysis of Fuzheng Jiedu Decoction on cervical cancer. Elect. J. Clin. Med. Lit. 61. doi: 10.16281/j.cnki.jocml.2020.14.058

Zhang, Y., Bie, W., Ma, Z. (2018). Clinical observation of Changfukang prescription combined with western medicine enema in the treatment of radiation enteritis with dampness-heat accumulation after radiotherapy for cervical cancer. Mod. J. Integr. Tradit. Chin. Western Med. 27, 3600–3603.

Zhang, Y., Zhang, J., Hong, Y. (2018). Clinical observation and study of mechanism of applying Wenyang Lishui decoction combined with intraperitone-al chemotherapy in the treatment of ovarian cancer patients with ascites hyperthermic perfusion. J. Sichuan Tradit. Chin. Med. 36, 157–160.

Zhao, C. (2013). Clinical Analysis of Related Factors and Therapeutic Effect of Pelvic Lymphocysts After Cervical Cancer Surgery. Liaoning University of Traditional Chinese Medicine, Shenyang, China.

Zhao, M. (2021). Effect of Jianpi Jiangni Tang combined with conventional Western medicine on adverse reactions after chemotherapy for cervical cancer. New Chin. Med. 53, 145–148. doi: 10.13457/j.cnki.jncm.2021.23.035

Zhu, P., Li, J. (2019). Effect of Fuzheng Zhuoma decoction combined with chemotherapy on T cell subsets, serum tumor marker expression and survival time in patients with advanced cervical cancer. Forum Tradit. Chin. Med. 34, 18–20. doi: 10.13913/j.cnki.41-1110/r.2019.01.008

Zhuang, Y., Chen, Y., Rui, W., Lu, Q., Luo, L. (2011). Analysis of curative effect of traditional Chinese medicine on radiation proctitis of cervical cancer. Guangdong Med. J. 32, 513–514. [doi](https://doi): 10.13820/j.cnki.gdyx.2011.04.041
